# The effect of coronary revascularization treatment timing on mortality in patients with stable ischemic heart disease in British Columbia

**DOI:** 10.1371/journal.pone.0303222

**Published:** 2024-10-24

**Authors:** Sean Hardiman, Guy Fradet, Lisa Kuramoto, Michael Law, Simon Robinson, Boris Sobolev

**Affiliations:** 1 School of Population and Public Health, University of British Columbia, Vancouver, Canada; 2 Faculty of Medicine, Department of Surgery, Division of Cardiovascular Surgery, University of British Columbia, Vancouver, Canada; 3 Vancouver Coastal Health Research Institute, Centre for Clinical Epidemiology and Evaluation, University of British Columbia, Vancouver, Canada; 4 Faculty of Medicine, Department of Medicine, Division of Cardiology, University of British Columbia, Vancouver, Canada; Saud Al-Babtain Cardiac Centre, SAUDI ARABIA

## Abstract

**Background:**

Prior research has shown that patients with stable ischemic heart disease who undergo delayed coronary artery bypass graft (CABG) surgery face higher mortality rates than those who receive CABG within the time recommended by physicians. However, this research did not account for percutaneous coronary intervention (PCI), a widely available alternative to delayed CABG in many settings. We sought to establish whether there was a difference in mortality between timely PCI and delayed CABG.

**Methods:**

We identified 25,520 patients 60 years or older who underwent first-time non-emergency revascularization for angiographically-proven, stable left main or multi-vessel ischemic heart disease in British Columbia between January 1, 2001, and December 31, 2016. We estimated unadjusted and adjusted mortality after index revascularization or last staged PCI for patients undergoing delayed CABG compared to timely PCI.

**Findings:**

After adjustment with inverse probability of treatment weights, at three years, patients who underwent delayed CABG had a statistically significant lower mortality compared with patients who received timely PCI (4.3% delayed CABG, 13.5% timely PCI; risk ratio 0.32, 95% CI 0.24–0.40).

**Interpretation:**

Patients who undergo CABG with delay have a lower risk of death than patients who undergo PCI within appropriate time. Our results suggest that patients who wish to receive CABG as their revascularization treatment will receive a mortality benefit over PCI as an alternative strategy.

## Introduction

In Canada, clinical need, resource allocation, and variation in demand determine how soon diagnosed coronary artery disease will be treated. Regional health authorities operate predominantly under a global budget funding model [1] that effectively caps the annual volume of procedures that a hospital can perform. Therefore, patients who require non-emergency revascularization by coronary artery bypass graft (CABG) surgery or percutaneous coronary intervention (PCI) may find their procedures are delayed during periods of higher demand for cardiac care or reduced supply of hospital services [2]. Access is further compromised during times of crisis, such as during the early waves of the COVID-19 pandemic throughout the country when non-emergency health care services were stopped and only emergency cases continued [3,4].

Prior research has shown that patients waiting for CABG benefit from earlier timing of treatment [5]. Multiple randomized clinical trials in patients with stable multi-vessel disease and left main disease have refined indications for CABG and PCI. Patients with multi-vessel or left-main coronary artery disease who do not need emergency treatment should consider CABG rather than PCI [6], due to lower mortality in some populations, fewer post-procedural myocardial infarctions, and a reduced need for repeat revascularization. However, none of these trials included patients with substantial delays in CABG treatment and evidence shows that mortality after CABG worsens when the surgery is delayed [5]. Moreover, PCI is considered a reasonable alternative to CABG. Therefore, we established our research question: do the proportions of long-term mortality differ between patients with stable multi-vessel or left main ischemic heart disease who have delayed CABG compared to those who have timely PCI? In other words, what would happen if patients who could only have CABG delivered below standard instead had PCI delivered to standard?

## Materials and methods

This study follows the Strengthening the Reporting of Observational Studies in Epidemiology (STROBE) guidelines for the reporting of observational cohort studies [7]. The University of British Columbia Clinical Research Ethics Board (Certificate H17-00505) provided ethical approval for this research.

We conducted a cohort study of prospectively collected data amongst all patients in British Columbia (BC) who underwent isolated CABG surgery or PCI for the treatment of coronary artery disease. We obtained diagnostic catheterization, PCI, and isolated CABG records from the provincial registries maintained by Cardiac Services BC (CSBC), a program of the Provincial Health Services Authority (Vancouver, BC). CSBC is responsible for the planning, funding, and quality of specialized tertiary cardiac services in the province, including cardiac surgery and interventional cardiology services. We used CSBC’s diagnostic catheterization, CABG, and PCI registry data to establish a single record that represents an episode of care which contains all events occurring from diagnostic catheterization through to revascularization. We linked these care episodes to the BC Ministry of Health’s Discharge Abstract Database (DAD), which contains hospitalization records, and the BC Vital Statistics Deaths File, which contains deaths data. Finally, we linked this data set to Population Data BC’s Central Demographics File, which contains demographic data for all study participants. In BC, the five cardiac centres are overseen by CSBC. CSBC structures, including annual quality reviews, bring together surgeons and interventional cardiologists from across the province. Each cardiac centre operates using a heart team model, though implementation varies amongst sites.

**Fig 1:**
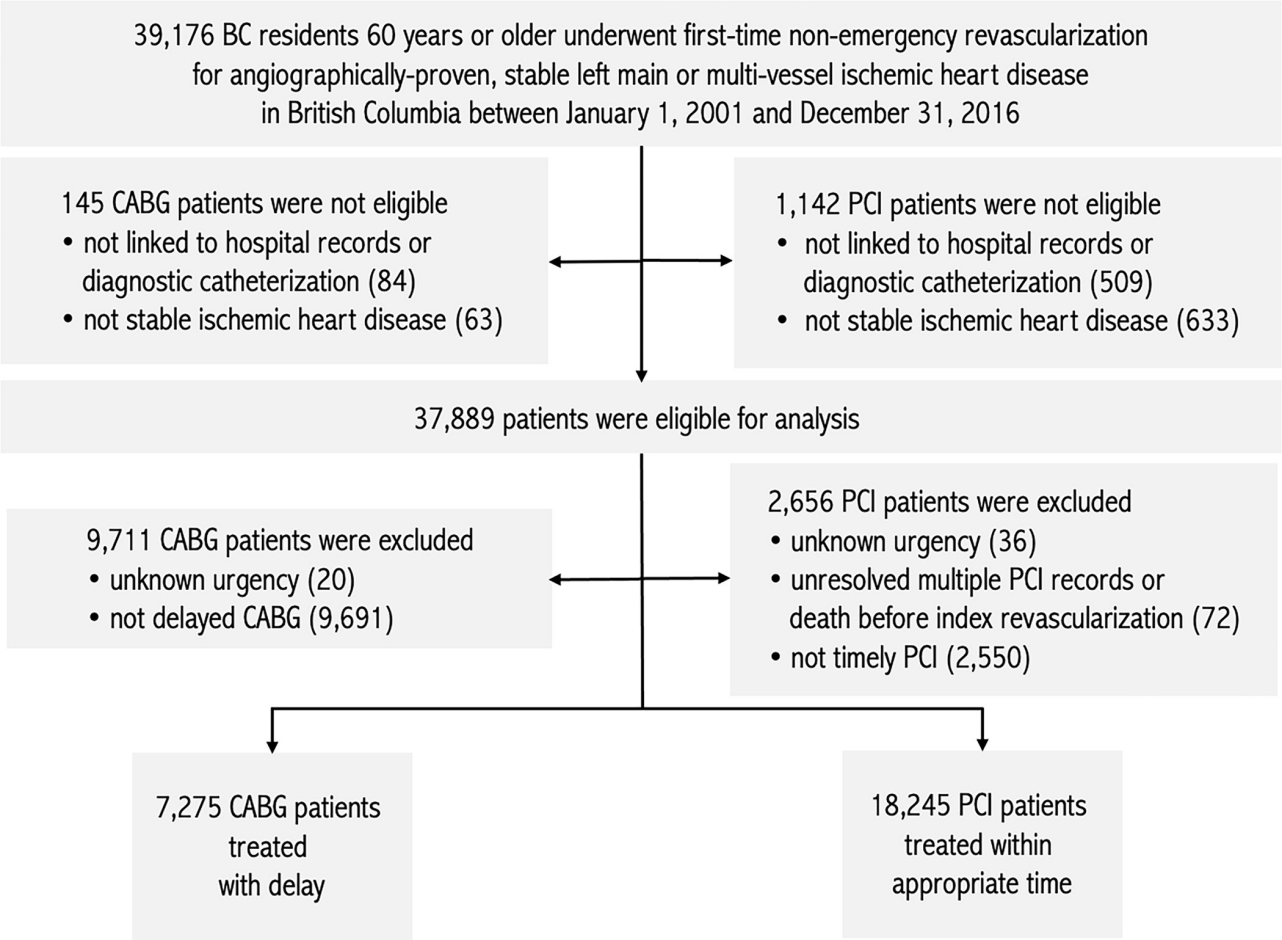
Flow chart for the study population.

The study consists of patients aged 60 years or older, who underwent non-emergency first-time revascularization for angiographically-proven, stable left main or multi-vessel ischemic heart disease in British Columbia, Canada, between January 1, 2001, and December 31, 2016 (Fig 1), criteria used in the ASCERT study [8]. We defined revascularization as either a PCI or an isolated CABG surgery. Patient age, extent of disease, and non-emergency status were identified using the Cardiac Services BC cardiac surgery and PCI registry data. Stable disease was identified using atherosclerotic heart disease code (ICD-10-CA I25.0, I25.1, I25.10; ICD-9 429.2 414.0) logged as type M (most responsible), type 1 (pre-admit comorbidity), type 2 (post-admit comorbidity), type 6 (proxy most responsible diagnosis), or types W, X, or Y (first, second, or third service transfers) in the DAD. The index event in this study is first-ever revascularization, by either PCI or CABG, within the study period of January 1, 2001, and December 31, 2016.

### Variables

#### Study variable

The study variable is treatment timing, operationalized as the time to coronary revascularization treatment and computed in calendar days. Based on treatment timing and the type of revascularization procedure received, patients were assigned to one of two study groups: delayed CABG or timely PCI. The time to treatment starts on the date when the need for revascularization is clinically established and the patient is ready, willing, and able to undergo revascularization. The time to treatment ends on the date the index revascularization procedure was performed. To establish intervals defining timely and delayed treatment, we used the Canadian Cardiovascular Society (CCS) recommended times [9] to define delayed CABG and timely PCI for semi-urgent and elective CABG and PCI patients, the First Minsters’ Meeting benchmarks [10] for urgent CABG, and CSBC benchmarks for urgent PCI patients (Table 1). In this analysis, appropriate treatment timing is defined by each of the three patient urgency categories. Table 1 defines the “appropriate time’ and ‘with delay’ time intervals for each urgency category”.

**Table 1:** Study group assignments by procedure type and urgency and treatment delay in days.

Procedure	Urgency	Timely Treatment		Delayed Treatment
		Interval Start	Interval End	Interval Start
CABG	Priority I	1 Day	7 Days	8+ Days
	Priority II	1 Day	14 Days	15+ Days
	Priority III	1 Day	42 Days	43+ Days
PCI	Urgent Inpatient	1 Day	5 Days	6+ Days
	Urgent Outpatient	1 Day	14 Days	15+ Days
	Elective	1 Day	42 Days	43+ Days

Dates were collected from CSBC registries for CABG and PCI, where triage coordinators recorded the date that the patient was booked for their procedure and the date their procedure occurred.

#### Outcome variable

The outcome variable is the time to death in days from any cause recorded in the BC Vital Statistics Deaths File. We followed patients from the time of index revascularization or last staged PCI until death, the end of the study, or three years’ follow-up, whichever came first. Due to data limitations, we developed a rule (S1 Appendix) to differentiate staged PCI from repeat revascularization in patients with multiple PCI records, based on Spitzer’s [11] criteria (S1 Appendix).

#### Additional variables

We used variables in the form in which they were received from the data stewards. Some concepts, such as comorbidities and clearance time, were operationalized by the study team from data already in the data set (S1 Appendix). Patients with a comorbidity identified as metastatic cancer process were grouped to the Metastatic Cancer variable. CABG patients whose CCS angina grade [12] was classified as Class 4A, 4B, or 4C in the CSBC cardiac surgery registry were grouped to Class 4.

### Statistical methods

We estimated the frequency and percentage of patients by characteristics and by treatment group. Groups were compared using a chi-square test for categorical variables and p-values for between group differences reported. We modelled cumulative mortality over three years using with a flexible parametric approach. This approach uses restricted cubic spline functions, also known as Royston-Parmar models, to model the baseline mortality over three years [13]. The main advantage of this model is that it provides the means to smoothly estimate the survival function, in contrast to the Cox model, where the baseline hazard function can be a noisy step function [13]. We then estimated the unadjusted cumulative mortality proportion over three years for each study group. Finally, we estimated risk ratios comparing the treatment groups. in mortality at three years. A risk ratio of less than one means the delayed CABG group had a lower risk of mortality at 3 years compared to the timely PCI group. A risk ratio of greater than one means the delayed CABG group had a higher risk of mortality at 3 years compared to the timely PCI group. We selected three years’ follow-up as the follow-up time, considering the variation in follow-up time used in randomized controlled trials of CABG versus PCI.

We estimated propensity scores [14] for the probability of belonging to each study group using logistic regression and, using those scores, calculated inverse probability of treatment weights [15], following the ASCERT approach [8]. Inverse probability of treatment weighting creates a synthetic cohort that utilizes all patient information, compared to other propensity score methods where this cannot be assured. Variables were selected starting with those used in ASCERT [8] and informed by a scoping review of the factors of mortality after CABG [16]. Each patient was weighted by the inverse of the probability of being assigned to their treatment group to adjust for differences between the two treatment groups. We compared the performance of the propensity score model by comparing the distribution of covariates and propensity scores before and after inverse probability weighting. Adjusted mortality estimates were obtained using an inverse probability weighted flexible parametric approach. Statistical analyses were performed using Stata 17 (College Station, TX). Flexible parametric models were constructed using *stpm2*, a Stata software package [17].

### Patient and public involvement

We consulted the Pacific Open-Heart Association (POHA) to inform development of our research question. POHA provides peer support to patients undergoing or who have undergone heart surgery in the Vancouver, BC area. They confirmed that BC patients often wait for CABG and that anxiety results when it isn’t known when a planned CABG will occur. In this study, we address the question that patients told us matters most: would it be better for CABG candidates to undergo PCI instead of facing an indeterminate length of time waiting for CABG?

### Data access

The authors first gained access to the data for research purposes on June 7, 2019, and had access to the data through April 25, 2024. The authors had no access to information that could identify individual participants during or after data collection.

## Results

### Setting and participants

We identified 39,176 British Columbia patients who met the selection criteria for our study (Fig 1).

We did not select patients for the analytical cohort if their revascularization record could not be linked to hospital records or their PCI record was for ad-hoc PCI, but the procedure could not be linked to a diagnostic catheterization (*n* = 591), or that their hospital records did not contain diagnosis codes indicative of stable ischemic heart disease (*n* = 696). 37,889 patients were eligible for analysis. We set aside patients if their procedure urgency could not be determined (*n* = 56), if patients with multiple PCI records were unresolved after applying the repeat revascularization algorithm or if there were errors in the administrative data set where date of death preceded date of revascularization (*n* = 72), if the patient received delayed PCI (*n* = 2,550), or if the patient received timely CABG (*n* = 9,711). 25,520 patients were available to be analyzed.

### Descriptive data

Table 2 shows the baseline characteristics of patients in the study cohort.

**Table 2:** Baseline characteristics of the patients.

	Unadjusted Data					Data Adjusted with Inverse Probability Weighting				
	Timely PCI (n = 18,245)		Delayed CABG (n = 7,275)		P-Value	Timely PCI (n = 26,376)		Delayed CABG (n = 22,813)		P-Value
	N	%	N	%		N	%	N	%	
Age*										
60–64	3,594	19.7%	1,609	22.1%	<0.001	5,150	19.5%	4,556	20.0%	0.17
65–69	3,849	21.1%	1,868	25.7%		5,862	22.2%	4,760	20.9%	
70–74	3,635	19.9%	1,827	25.1%		5,811	22.0%	5,950	26.1%	
75–79	3,361	18.4%	1,355	18.6%		4,791	18.2%	4,225	18.5%	
> = 80	3,806	20.9%	616	8.5%		4,762	18.1%	3,322	14.6%	
Sex										
Male	12,718	69.7%	5,994	82.4%	<0.001	19,389	73.5%	16,323	71.6%	0.49
Female	5,527	30.3%	1,281	17.6%		6,987	26.4%	6,489	28.4%	
Body Mass Index*										
<18.5	237	1.3%	43	0.6%	<0.001	298	1.1%	130	0.6%	0.52
≥18.5 and <25	5,380	29.5%	1,897	26.1%		7,583	28.8%	6,718	29.4%	
≥25 and <30	7,886	43.2%	3,173	43.6%		11,452	43.4%	9,983	43.8%	
>30	4,625	25.3%	2,046	28.1%		6,857	26.0%	5,777	25.3%	
Missing	117	0.6%	116	1.6%		186	0.7%	205	0.9%	
Extent of Disease										
Double Vessel Disease	10,076	55.2%	543	7.5%	<0.001	10,575	40.1%	8,350	36.6%	0.18
Triple Vessel Disease	7,290	40.0%	4,587	63.1%		11,643	44.1%	11,151	48.9%	
Left Main Disease	879	4.8%	2,145	29.5%		4,158	15.8%	3,311	14.5%	
Ejection Fraction†										
EF <30%	697	3.8%	268	3.7%	<0.001	984	3.7%	830	3.6%	0.70
EF ≥30% and ≤50%	3,389	18.6%	1,965	27.0%		5,765	21.9%	4,550	19.9%	
EF >50%	10,410	57.1%	4,496	61.8%		15,244	57.8%	13,385	58.7%	
Missing	3,749	20.5%	546	7.5%		4,383	16.6%	4,047	17.7%	
Serum Creatinine (μmol/L)*										
<60	775	4.2%	219	3.0%	<0.001	977	3.7%	1,281	5.6%	0.47
60≥ and <80	4,380	24.0%	1,525	21.0%		6,149	23.3%	5,308	23.3%	
80≥ and <99	6,400	35.1%	2,470	34.0%		9,332	35.4%	8,134	35.7%	
≥100	6,024	33.0%	2,320	31.9%		8,562	32.5%	6,813	29.9%	
Unknown	666	3.7%	741	10.2%		1,355	5.1%	1,276	5.6%	
Canadian Cardiovascular Society Angina Class*										
None	715	3.9%	389	5.3%	<0.001	1,082	4.1%	1,161	5.1%	0.12
Class 1	661	3.6%	347	4.8%		970	3.7%	1,020	4.5%	
Class 2	2,882	15.8%	1,785	24.5%		4,872	18.5%	4,473	19.6%	
Class 3	1,452	8.0%	2,730	37.5%		4,724	17.9%	4,030	17.7%	
Class 4	11,450	62.8%	1,494	20.5%		12,821	48.6%	10,113	44.3%	
Atypical	314	1.7%	68	0.9%		377	1.4%	208	0.9%	
Missing	771	4.2%	462	6.4%		1,529	5.8%	1,807	7.9%	
Prior Acute Myocardial Infarction*										
Yes	3,479	19.1%	2,516	34.6%	<0.001	6,843	25.9%	5,993	26.3%	0.98
Unknown	4,385	24.0%	2,109	29.0%		6,616	25.1%	5,654	24.8%	
Smoking Status*										
Never	7,094	38.9%	2,626	36.1%	<0.001	9,620	36.5%	8,798	38.6%	0.34
Current/Now	2,283	12.5%	621	8.5%		2,979	11.3%	2,181	9.6%	
Former/Quit	8,226	45.1%	3,086	42.4%		12,129	46.0%	10,054	44.1%	
Unknown	642	3.5%	942	12.9%		1,648	6.2%	1,780	7.8%	
Comorbidities										
Atrial Fibrillation or Atrial Flutter	1,421	7.8%	2,051	28.2%	<0.001	3,210	12.2%	3,236	14.2%	0.14
Cardiac Dysrhythmias§	728	4.0%	351	4.8%	0.001	1,083	4.1%	1,477	6.5%	0.05
Cerebrovascular Disease	428	2.3%	379	5.2%	<0.001	906	3.4%	1,251	5.5%	0.27
Chronic Pulmonary Disease	911	5.0%	341	4.7%	0.31	1,465	5.6%	982	4.3%	0.23
Congestive Heart Failure	2,109	11.6%	958	13.2%	<0.001	3,474	13.2%	2,496	10.9%	0.09
Connective Tissue Disease	238	1.3%	93	1.3%	0.87	356	1.3%	280	1.2%	0.78
Diabetes	4,986	27.3%	2,908	40.0%	<0.001	8,484	32.2%	7,416	32.5%	0.88
Hypertension	9,102	49.9%	4,450	61.2%	<0.001	14,237	54.0%	11,814	51.8%	0.39
Hypertensive Heart Disease	35	0.2%	18	0.2%	0.38	149	0.6%	48	0.2%	0.21
Liver Disease	53	0.3%	19	0.3%	0.69	105	0.4%	72	0.3%	0.67
Metastatic Cancer	496	2.7%	168	2.3%	0.06	662	2.5%	662	2.9%	0.67
Peripheral Vascular Disease	715	3.9%	460	6.3%	<0.001	1,404	5.3%	1,064	4.7%	0.55
Pneumonia	522	2.9%	287	3.9%	<0.001	1,088	4.1%	776	3.4%	0.50
Renal Disease	1,240	6.8%	711	9.8%	<0.001	1,895	7.2%	1,770	7.8%	0.59
Ulcer Disease	104	0.6%	104	1.4%	<0.001	179	0.7%	494	2.2%	0.01
Calendar Period of Index Revascularization										
2001	949	5.2%	484	6.7%	<0.001	1,532	5.8%	1,901	8.3%	0.26
2002	1,101	6.0%	684	9.4%		1,766	6.7%	2,088	9.2%	
2003	1,210	6.6%	714	9.8%		2,023	7.7%	2,180	9.6%	
2004	1,224	6.7%	544	7.5%		1,826	6.9%	1,612	7.1%	
2005	1,166	6.4%	503	6.9%		1,844	7.0%	1,499	6.6%	
2006	1,100	6.0%	512	7.0%		1,688	6.4%	1,260	5.5%	
2007	1,252	6.9%	485	6.7%		1,690	6.4%	1,353	5.9%	
2008	1,266	6.9%	373	5.1%		1,797	6.8%	1,256	5.5%	
2009	1,342	7.4%	238	3.3%		1,790	6.8%	1,592	7.0%	
2010	1,387	7.6%	263	3.6%		1,639	6.2%	945	4.1%	
2011	1,325	7.3%	250	3.4%		1,576	6.0%	1,084	4.8%	
2012	1,122	6.1%	347	4.8%		1,467	5.6%	1,094	4.8%	
2013	897	4.9%	467	6.4%		1,470	5.6%	1,373	6.0%	
2014	939	5.1%	455	6.3%		1,434	5.4%	1,295	5.7%	
2015	905	5.0%	421	5.8%		1,296	4.9%	940	4.1%	
2016	1,060	5.8%	535	7.4%		1,537	5.8%	1,339	5.9%	
Hospital Type										
Metropolitan	14,197	77.8%	5,396	74.2%	<0.001	20,531	77.8%	16,981	74.4%	0.17
Urban	4,048	22.2%	1,879	25.8%		5,845	22.2%	5,831	25.6%	
Clearance Time Category¶										
1 Week	14,410	79.0%	3,052	42.0%	<0.001	17,410	66.0%	14,689	64.4%	0.66
2 Weeks	2,387	13.1%	1,910	26.3%		4,836	18.3%	4,332	19.0%	
3 or More Weeks	1,448	7.9%	2,313	31.8%		4,130	15.7%	3,791	16.6%	
Neighborhood Income Decile										
Lowest Decile	1,956	10.7%	707	9.7%	0.22	2,664	10.1%	2,417	10.6%	0.87
2nd Decile	1,925	10.6%	756	10.4%		2,814	10.7%	2,089	9.2%	
3rd Decile	1,822	10.0%	742	10.2%		2,742	10.4%	2,427	10.6%	
4th Decile	1,907	10.5%	784	10.8%		2,934	11.1%	2,790	12.2%	
5th Decile	1,778	9.7%	759	10.4%		2,374	9.0%	2,030	8.9%	
6th Decile	1,760	9.6%	645	8.9%		2,392	9.1%	2,249	9.9%	
7th Decile	1,699	9.3%	694	9.5%		2,459	9.3%	2,097	9.2%	
8th Decile	1,755	9.6%	708	9.7%		2,774	10.5%	2,131	9.3%	
9th Decile	1,698	9.3%	706	9.7%		2,421	9.2%	1,957	8.6%	
Highest Decile	1,679	9.2%	673	9.3%		2,346	8.9%	2,360	10.3%	
Unknown	266	1.5%	101	1.4%		457	1.7%	265	1.2%	

* At the time of revascularization.

† Ejection Fraction at the time of revascularization; if missing, at the time of diagnostic catheterization.

§ Excluding atrial fibrillation and atrial flutter.

¶ Clearance time is the hypothetical time within which the wait list would be cleared at maximum weekly service capacity if there were no new arrivals.

Before adjustment with inverse probability weighting, the patients undergoing delayed CABG were, compared to patients undergoing timely PCI, had higher proportions of male sex, a BMI >30, triple vessel disease, left main disease, and an ejection fraction ≤50%. The delayed CABG group had significantly higher proportions of atrial fibrillation or atrial flutter, congestive heart failure, diabetes, hypertension, and renal disease, compared to timely PCI. The timely PCI group had higher proportions of double-vessel disease and Canadian Cardiovascular Society (CCS) Angina Class 4. Most patients, regardless of study group, were treated in metropolitan hospitals. Clearance time is shorter amongst patients treated with timely PCI compared to delayed CABG. Proportions of neighborhood income decile are balanced throughout the study cohort. Of the patients who underwent PCI, 48.1% received Bare-Metal Stents (BMS), 4.5% received a combination of BMS and Drug-Eluting Stents (DES) and 42.8% received only DES. Of the patients who underwent CABG, 8.5% received only a saphenous vein graft, 71.6% received a single arterial graft, 16.3% received a double arterial graft, and 3.4% received a triple arterial graft. The mean waiting time for the timely PCI group was 11.9 days and the mean waiting time for the delayed CABG group was 71.5 days (S1 Appendix).

As expected, patients in the timely PCI group had a lower probability of being selected for delayed CABG than did those in the CABG group. However, all patients had a positive probability of being assigned to either CABG or PCI, consistent with results of comparative effectiveness studies of CABG and PCI published elsewhere [8].

### Outcome data and main results

**Fig 2:**
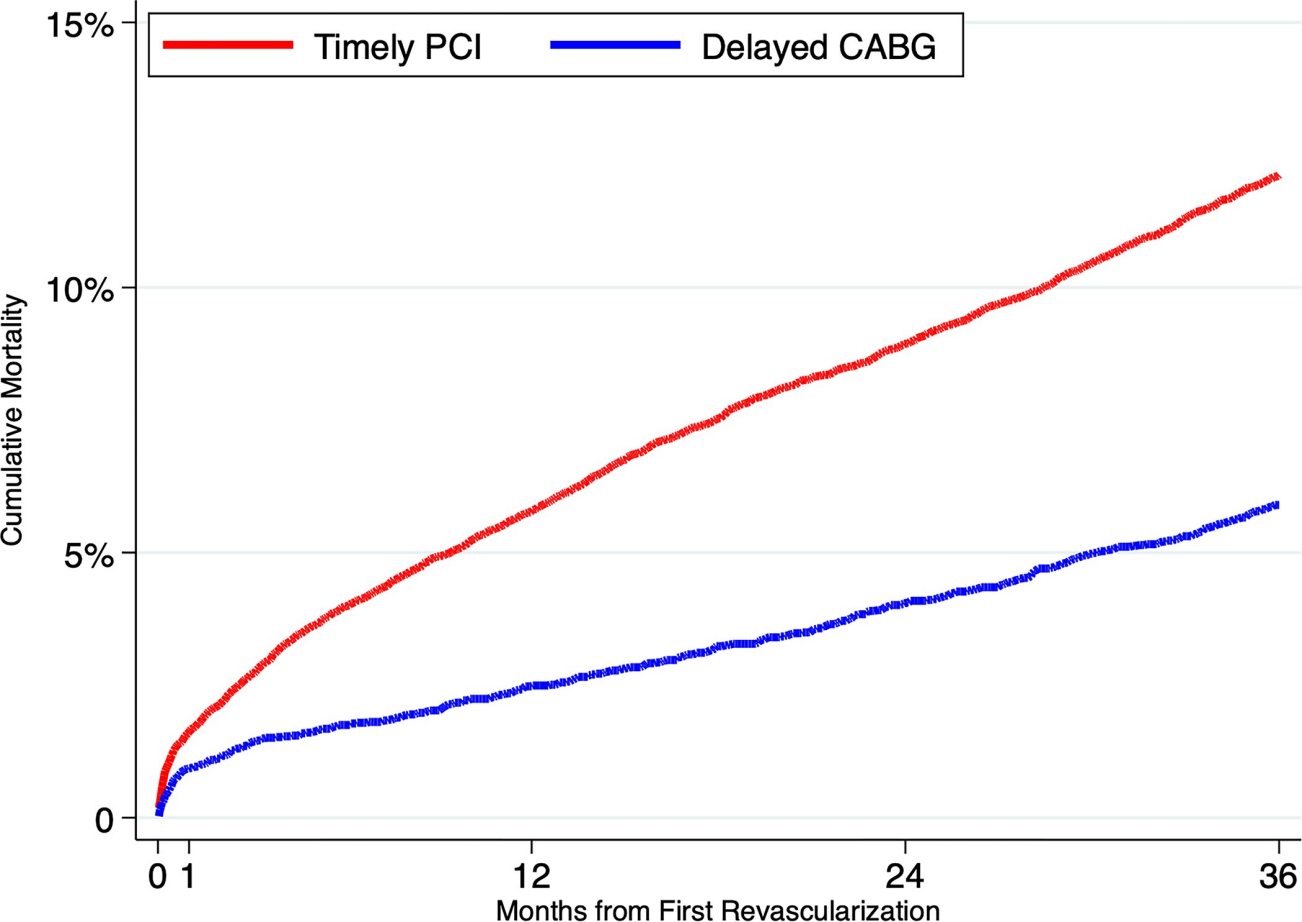
Cumulative mortality in the CABG and PCI populations, from an unadjusted analysis.

Unadjusted failure curves are shown in Fig 2; unadjusted cumulative mortality and risk ratios are shown in Table 3.

**Table 3:** Rates of mortality (percent), risk ratios, and 95% confidence intervals in the delayed CABG and timely PCI populations, from an unadjusted analysis.

	30 Days	1 Year	2 Years	3 Years
Delayed CABG	0.7 (0.6, 0.9)	2.6 (2.3, 2.9)	4.2 (3.8, 4.6)	5.9 (5.3, 6.4)
Timely PCI	1.5 (1.4, 1.7)	5.8 (5.4, 6.1)	8.9 (8.5, 9.3)	12.1 (11.6, 12.6)
Risk Ratio for Delayed CABG	0.48 (0.36, 0.60)	0.45 (0.39, 0.52)	0.47 (0.42, 0.52)	0.48 (0.43, 0.53)

At 30 days, there was a significant difference in unadjusted mortality between the groups (0.7% in the delayed CABG group compared with 1.5% in the timely PCI group). The 1-year unadjusted mortality rate was 2.6% in the delayed CABG group and 5.8% in the timely PCI group. The 2-year unadjusted mortality rate was 4.2% in the delayed CABG group and 8.9% in the timely PCI group. The 3-year unadjusted mortality rate was 5.9% in the delayed CABG group and 12.1% in the timely PCI group (risk ratio [RR] 0.48, 95% confidence interval [CI], 0.43–0.53).

**Fig 3:**
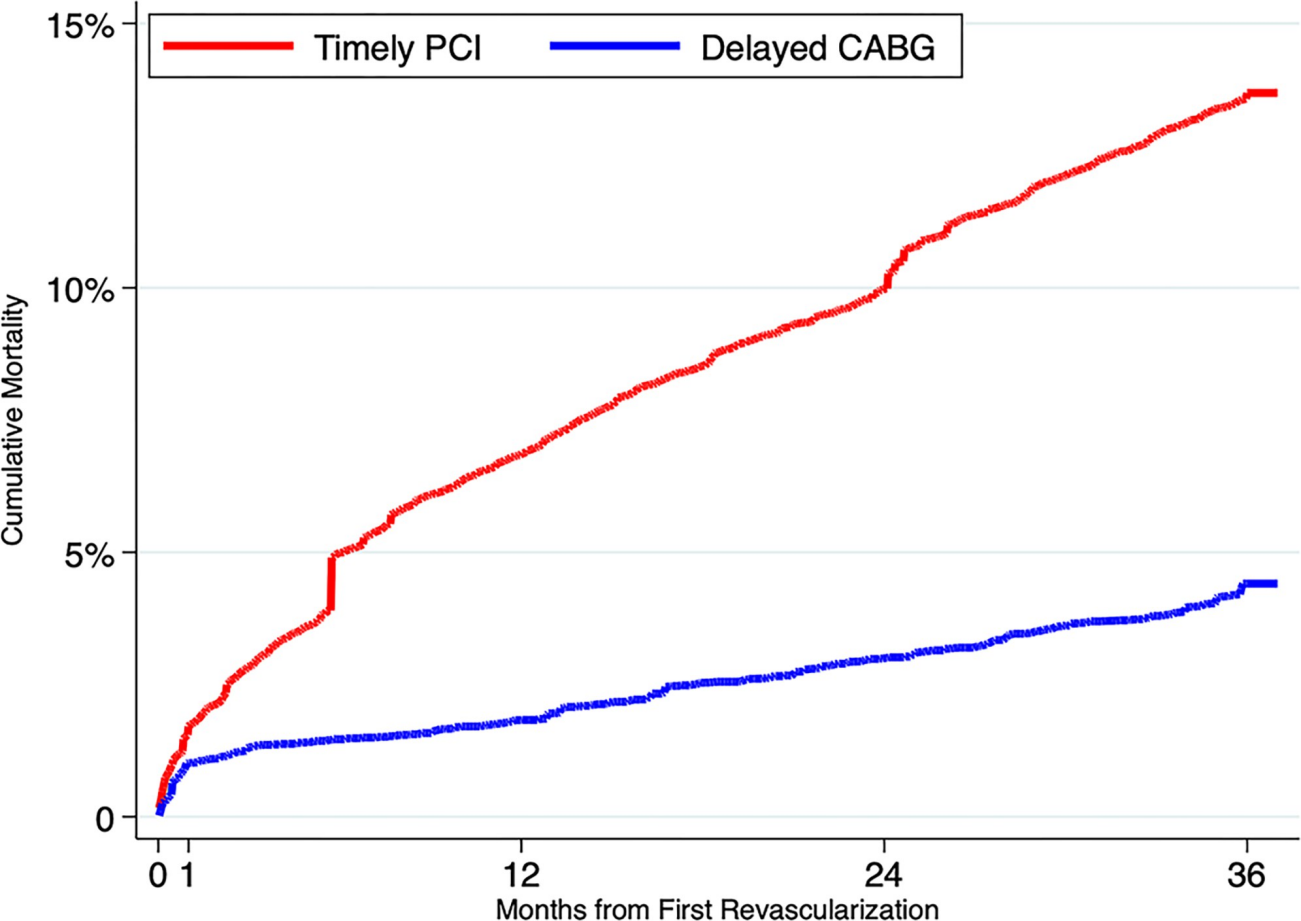
Cumulative mortality in the CABG and PCI populations, from an analysis adjusted with the use of inverse probability of treatment weighting.

Failure curves adjusted with inverse probability of treatment weighting are shown in Fig 3; adjusted cumulative mortality and risk ratios are shown in Table 4.

**Table 4:** Rates of mortality (percent), risk ratios, and 95% confidence intervals in the delayed CABG and timely PCI populations, from an adjusted analysis.

	30 Days	1 Year	2 Years	3 Years
Delayed CABG	0.7 (0.3, 1.0)	2.1 (1.5, 2.8)	3.2 (2.5, 3.9)	4.3 (3.4, 5.1)
Timely PCI	1.5 (1.3, 1.8)	6.7 (5.0, 8.4)	10.1 (8.0, 12.3)	13.5 (11.4, 15.5)
Risk Ratio for Delayed CABG	0.45 (0.20, 0.69)	0.32 (0.20, 0.44)	0.32 (0.23, 0.41)	0.32 (0.24, 0.40)

At 30 days, there was a significant difference in adjusted mortality between the groups (0.7% in the delayed CABG group compared with 1.5% in the timely PCI group, RR 0.32; 95% CI 0.24–0.40). The 1-year adjusted mortality was 2.1% in the delayed CABG group and 6.7% in the timely PCI group. The 2-year adjusted mortality was 3.2% in the delayed CABG group compared with 10.1% in the timely PCI group. The 3-year adjusted mortality rate was 4.3% in the delayed CABG group and 13.5% in the timely PCI group (risk ratio 0.32, 95% CI, 0.24 to 0.40).

## Discussion

### Key results

This study used data from multiple population-based registries and databases to evaluate the effectiveness of delayed CABG as compared with timely PCI. In this study, we found that amongst British Columbia patients 60 aged years or older, who underwent non-emergency first-time revascularization for angiographically-proven, stable left main or multi-vessel ischemic heart disease in British Columbia, between January 1, 2001, and December 31, 2016, there was a significant difference in both unadjusted mortality and mortality adjusted using inverse probability of treatment weights at 30 days, one year, two years, and three years. When the study cohort was stratified into three time periods, results were consistent with the results observed in the primary analysis (S1 Appendix).

Our findings should be considered in the context of results from other studies. There have been seven randomized, controlled trials comparing CABG with balloon angioplasty, eleven comparing CABG with PCI and stenting in patients with multi-vessel disease and six comparing CABG with PCI and stenting in specifically in patients with left-main disease. A survival advantage for CABG was noted in the Stent or Surgery (SoS) trial at two years [18] and sustained at six years [19]. In the ARTS-I trial, where BMS were used, a survival advantage at five years was not found [20]. However, the ARTS-II trial, which compared the ARTS-I CABG cohort to a new PCI cohort where patients received DES, did find a statistically significant difference in mortality. The FREEDOM trial, where diabetics with multivessel disease were treated with CABG compared to PCI with DES also found a statistically significant difference in mortality at five years [21], and again at seven years [22], consistent with the subgroup analysis reported by the BARI Investigators [23], but inconsistent with the SYNTAX diabetes subgroup study that showed no statistical difference [24], though a trend to better outcomes with CABG was observed. In the SYNTAX study, no difference in all-cause mortality was observed at three years, however, a statistically significant benefit was found for CABG patients with triple-vessel disease [25]. The benefits for patients with triple-vessel disease were sustained in the ten-year SYNTAX extension study [26].

Of note is the finding of a difference in mortality at 30 days of index revascularization or last staged PCI. Randomized controlled trials and observational studies have shown higher mortality in patients undergoing CABG during the first 30 days compared to patients undergoing PCI, attributed to increased risk of death in the immediate post-operative period. We selected only patients with stable ischemic heart disease, who would be expected to have lower surgical risk than patients with more unstable disease. This may account for lower early mortality observed in this study. BC also has a long-standing cardiac surgery quality oversight program, delivered by CSBC in collaboration with the cardiac surgery community. Annual reporting on hospital and surgeon mortality at 30 days and 1 year during the study period may have contributed to improvements in the structures and processes associated with care quality that could contribute to lower mortality.

Observational studies also inform our understanding of these results. The ASCERT study [8] examined the comparative effectiveness of CABG versus PCI in Medicare patients using patient selection criteria and analytical methods common to ours. While no significant difference in mortality was found after one year, lower mortality was observed with CABG compared to PCI at four years, similar to our results. Similar findings were recently reported by Mehaffey and colleagues using contemporary CABG and PCI techniques in Medicare patients [27]. Our findings are consistent with those of a recent systematic review [28] included 23 studies comparing CABG with PCI. CABG was associated with better survival during follow-up in 17 studies and no significant difference between treatments in six, with no study favouring PCI. A recent meta-analysis reported similar results in patients who received CABG compared to PCI with DES [29].

Prior research also shows that the incidence of adverse events on CABG waitlists is low in the non-emergency population. Specifically, the risk of death has been shows to be approximately 1% [30] in one study, with a death rate 0.5 per 1000 patient-weeks in semi-urgent patients and 0.6 per 1000 patient-weeks in non-urgent patients [31]. The emergency surgery rate in the latter study was 1.2 per 1000 patient-weeks in the non-urgent group, while the rate was 2.1 per 1000 patient weeks in the semi-urgent group. Given the low frequency of these adverse events while waiting in the non-emergency population, we would not expect our conclusions to change because of events occurring prior to index revascularization, the time where we begin to measure outcomes in this analysis. We are unaware of any studies that examine the effect of waiting for PCI. This is expected given that PCI is generally available both in Canada and in the United States within relatively short time periods. Our study uses timing intervals recommended by Canadian Cardiovascular Society physicians [9]. Thus, in the case of PCI, a very low event rate would be expected in stable patients given the consensus guidance to treat the lowest acuity stable patients within 42 days.

We conducted a sensitivity analysis to investigate the potential for unmeasured confounding. Using the using the methodology proposed by VanderWeele and Ding [32], we calculated the E-value for the analysis. The E-value is the minimum strength of association, on the risk ratio scale, that an unmeasured confounder would need to have, with both the treatment and outcome, to fully explain away a specific association, conditional on the measured covariates. A large E-value suggests considerable unmeasured confounding would be needed to explain away an effect estimate, while a small one suggests little unmeasured confounding would be needed. We determined that the observed risk ratio of 0.32 could only be explained by an unmeasured confounder with a risk ratio of at least 5.7-fold each (95% CI closest to the null risk ratio, 4.4), beyond the measured covariates. Therefore, it seems unlikely that a single unknown factor could have an effect sufficiently large enough to account, on its own, for the observed difference in mortality between the treatment groups.

While we hypothesized that at some point, patients with stable disease would benefit from changing therapies to PCI, this was not observed. It is known that in BC during the study period that significant CSBC quality oversight structures were in place for CABG whereas for PCI, these were only introduced in the latter third of the study period. Quality review and quality improvement mechanisms may have contributed to lower mortality in the CABG group and possibly higher mortality in the PCI group.

These results are noteworthy in that they demonstrate that amongst this patient population, the benefits of CABG do not appear to be attenuated by delay compared to PCI. For physicians who must advise patients on treatment strategies in the context of scarce resources, these results suggest that PCI as an alternative revascularization strategy may not be indicated if a reduced risk of mortality is desired. Patients can know that waiting for CABG may have benefits over PCI. Policymakers should interpret these results in the context of past CABG research, which shows benefit to earlier timing of treatment [5].

### Limitations

This study has limitations. First, this study utilizes observational data as the basis for its analysis and conclusions. While took steps to model our design after a randomized controlled trial using the guidance of Cochran [33] and Rosenbaum [34], it’s possible these efforts were insufficient. It’s possible that group comparability was not the same in this study as in a randomized trial given the many factors, both conscious and unconscious, that may affect a revascularization method selection. Second, it’s possible unmeasured confounders affected our results. While we used inverse probability of treatment weights to balance differences in patient and health system factors and conducted a sensitivity analysis, it is still possible that unmeasured confounders affected these results. Third, we studied patients who underwent treatment between 2001 and 2016, during which stent technology evolved significantly, and the use of antiplatelet therapy evolved significantly. While we accounted for this by adjusting for calendar year of procedure, this may not have been sufficient to address the effect of time on outcomes. Fourth, we were limited by data available from CSBC in how we could establish extent of disease. While revascularization appropriate use criteria [35] suggest the use of SYNTAX scores to differentiate eligibility for CABG or PCI, this data is not routinely collected in BC. While SYNTAX scores are thought to have limited utility due to inter-rater variability [6], the absence of this data limited our ability to stratify our patient groups to match those proposed in appropriate use criteria. Fifth, the dataset lacked specific reasons for delaying CABG. Previous studies have linked variations in time to procedure to the weekly number of patients on the CABG waiting list and the weekly number of urgent cases [36]; therefore, we accounted for these factors by creating a clearance time variable that was included in the propensity score model. Sixth, clinical presentation data was not consistently available during the study period, so we used diagnosis codes to identify patients with stable disease and did not select any patient with an ‘emergency’ priority for our study cohort. These efforts may not have completely excluded patients with more serious acuity who were not eligible for CABG and instead treated with PCI. Finally, this study was performed in a publicly funded health system in Canada where access to care is limited by funded and operationalized capacity. Thus, its results may not generalize to other health systems where capacity is not a factor that constrains access to care.

### Interpretation

Our results suggest that there is evidence that the treatment benefit of CABG surgery is not attenuated because of a delay in treatment when compared to PCI provided within appropriate time.

### Generalizability

As a population-based study, our results can be generalized to similar populations as those selected for this study. Our results can also be generalized to cardiac services systems with structures and processes that align with those that found in BC. Caution should be taken in applying these results to other populations and systems.

## Conclusion

In summary, this study used data from the CSBC diagnostic catheterization, PCI, and CABG registries, linking to the DAD, the BC Vital Statistics Deaths File and Population Health Data BC’s Central Demographics File to assess the comparative effectiveness of timely PCI and delayed CABG. We found that amongst patients older than 60 years of age with stable, multi-vessel or left-main ischemic heart disease that did not require emergency treatment that there was a statistically significant short-term and long-term survival advantage for patients who underwent delayed CABG compared to those who had timely PCI. Patients who face extended waiting times for CABG should be aware of these benefits before choosing PCI as an alternative revascularization strategy. Given these findings and the continued evolution of both CABG and PCI procedures, further research on the effects of delay is indicated.

## Supporting information

S1 Appendix (DOCX)

## Abbreviations

BC: British Columbia
BMS: Bare-Metal Stent
CABG: Coronary Artery Bypass Graft
CCS: Canadian Cardiovascular Society
CSBC: Cardiac Services BC
DAD: Discharge Abstract Database
DES: Drug-Eluting Stent
PCI: Percutaneous Coronary Intervention
POHA: Pacific Open Heart Association
STROBE: Strengthening the Reporting of Observational Studies in Epidemiology

## References

[1] Sutherland JM. Hospital payment mechanisms: An overview and options for Canada. Ottawa, ON: Canadian Health Services Research Foundation; 2011 Mar pp. 1–26. Available: HTTPS://POLICYCOMMONS.NET/ARTIFACTS/1201257/HOSPITAL-PAYMENT-MECHANISMS/1754377/.

[2] Sher J. “Limited system-wide capacity” to blame for cancelling Londoner’s bypass four times, hospital says. 22 Feb 2018. Available: HTTPS://LFPRESS.COM/NEWS/LOCAL-NEWS/NO-BED-NO-HEART-SURGERY.

[3] British Columbia Ministry of Health. Joint statement on B.C.’s COVID-19 response and latest updates. 2020. Available: HTTPS://NEWS.GOV.BC.CA/RELEASES/2020HLTH0086-000499.

[4] Anderson M. Ramp down of elective surgeries and non-emergent/non-urgent acute care activities. 8 Apr 2021 [cited 24 May 2021]. Available: HTTPS://WWW.CORHEALTHONTARIO.CA/OH-RAMP-DOWN-MEMO-APRIL-8-2021.PDF.

[5] Sobolev BG, Fradet G, Kuramoto L, Rogula B. An observational study to evaluate 2 target times for elective coronary bypass surgery. Med Care. 2012;50: 611–9. doi: 10.1097/MLR.0b013e31824deed2 22525613

[6] Lawton JS, Tamis-Holland JE, Bangalore S, Bates ER, Beckie TM, Bischoff JM, et al. 2021 ACC/AHA/SCAI Guideline for Coronary Artery Revascularization A Report of the American College of Cardiology/American Heart Association Joint Committee on Clinical Practice Guidelines. J Am Coll Cardiol. 2021. doi: 10.1016/j.jacc.2021.09.006 34895950

[7] Elm E von, Altman DG, Egger M, Pocock SJ, Gøtzsche PC, Vandenbroucke JP, et al. The Strengthening the Reporting of Observational Studies in Epidemiology (STROBE) statement: guidelines for reporting observational studies. American College of Physicians; 2007. 10.1136/bmj.39335.541782.AD PMC2034723 17947786

[8] Weintraub WS, Grau-Sepulveda MV, Weiss JM, O’Brien SM, Peterson ED, Kolm P, et al. Comparative effectiveness of revascularization strategies. The New England journal of medicine. 2012;366: 1467–1476. doi: 10.1056/NEJMoa1110717 22452338 PMC4671393

[9] Graham MM, Knudtson ML, O’Neill BJ, Ross DB, Canadian Cardiovascular Society Access to Care Working Group. Treating the right patient at the right time: Access to cardiac catheterization, percutaneous coronary intervention and cardiac surgery. Canadian Journal of Cardiology. 2006;22: 679–683. 16801998 10.1016/s0828-282x(06)70936-9 PMC2560560

[10] Government of Canada. First ever common benchmarks will allow Canadians to measure progress in reducing wait times. 2005. Available: HTTPS://WWW.CANADA.CA/EN/NEWS/ARCHIVE/2005/12/FIRST-EVER-COMMON-BENCHMARKS-ALLOW-CANADIANS-MEASURE-PROGRESS-REDUCING-WAIT-TIMES.HTML.

[11] Spitzer E, McFadden E, Vranckx P, Vries T de, Ren B, Collet C, et al. Defining staged procedures for percutaneous coronary intervention trials: A guidance document. JACC Cardiovascular interventions. 2018;11: 823–832. doi: 10.1016/j.jcin.2018.03.044 29747912

[12] Campeau L. Letter: Grading of angina pectoris. Circulation. 1976;54: 522–523. 947585

[13] Royston P, Parmar MKB. Flexible parametric proportional‐hazards and proportional‐odds models for censored survival data, with application to prognostic modelling and estimation of treatment effects. Statist Med. 2002;21: 2175–2197. doi: 10.1002/sim.1203 12210632

[14] Rosenbaum PR, Rubin DB. The central role of the propensity score in observational studies for causal effects. Biometrika. 1983;70: 41–55. Available: HTTP://BIOMET.OXFORDJOURNALS.ORG/CONTENT/70/1/41.ABSTRACT.

[15] Austin PC, Stuart EA. Moving towards best practice when using inverse probability of treatment weighting (IPTW) using the propensity score to estimate causal treatment effects in observational studies. Stat Med. 2015;34: 3661–3679. doi: 10.1002/sim.6607 26238958 PMC4626409

[16] Hardiman SC, Villan YFV, Conway JM, Sheehan KJ, Sobolev B. Factors affecting mortality after coronary bypass surgery: a scoping review. J Cardiothorac Surg. 2022;17: 45. doi: 10.1186/s13019-022-01784-z 35313895 PMC8935749

[17] Lambert PC, Royston P. Further development of flexible parametric models for survival analysis. Stata J. 2009;9: 265–290. doi: 10.1177/1536867x0900900206

[18] SoS Investigators. Coronary artery bypass surgery versus percutaneous coronary intervention with stent implantation in patients with multivessel coronary artery disease (the Stent or Surgery trial): a randomised controlled trial. Lancet. 2002;360: 965–970. doi: 10.1016/S0140-6736(02)11078-6 12383664

[19] Booth J, Clayton T, Pepper J, Nugara F, Flather M, Sigwart U, et al. Randomized, controlled trial of coronary artery bypass surgery versus percutaneous coronary intervention in patients with multivessel coronary artery disease: six-year follow-up from the Stent or Surgery Trial (SoS). Circulation. 2008;118: 381–388. doi: 10.1161/CIRCULATIONAHA.107.739144 18606919

[20] Serruys PW, Ong ATL, Herwerden LA van, Sousa JE, Jatene A, Bonnier JJRM, et al. Five-year outcomes after coronary stenting versus bypass surgery for the treatment of multivessel disease: the final analysis of the Arterial Revascularization Therapies Study (ARTS) randomized trial. Journal of the American College of Cardiology. 2005;46: 575–581. doi: 10.1016/j.jacc.2004.12.082 16098418

[21] Farkouh ME, Domanski M, Sleeper LA, Siami FS, Dangas G, Mack M, et al. Strategies for multivessel revascularization in patients with diabetes. The New England journal of medicine. 2012;367: 2375–2384. doi: 10.1056/NEJMoa1211585 23121323

[22] Farkouh ME, Domanski M, Dangas GD, Godoy LC, Mack MJ, Siami FS, et al. Long-term survival following multivessel revascularization in patients with diabetes (FREEDOM follow-on study). Journal of the American College of Cardiology. 2018. doi: 10.1016/j.jacc.2018.11.001 30428398 PMC6839829

[23] The BARI Investigators. Influence of diabetes on 5-year mortality and morbidity in a randomized trial comparing CABG and PTCA in patients with multivessel disease: The Bypass Angioplasty Revascularization Investigation (BARI). Circulation. 1997;96: 1761–1769. doi: 10.1161/01.cir.96.6.1761 9323059

[24] Kappetein AP, Head SJ, Morice M-C, Banning AP, Serruys PW, Mohr F-W, et al. Treatment of complex coronary artery disease in patients with diabetes: 5-year results comparing outcomes of bypass surgery and percutaneous coronary intervention in the SYNTAX trial. European journal of cardio-thoracic surgery: official journal of the European Association for Cardio-thoracic Surgery. 2013;43: 1006–1013. doi: 10.1093/ejcts/ezt017 23413014

[25] Kappetein AP, Feldman TE, Mack MJ, Morice M-C, Holmes DR, Ståhle E, et al. Comparison of coronary bypass surgery with drug-eluting stenting for the treatment of left main and/or three-vessel disease: 3-year follow-up of the SYNTAX trial. European heart journal. 2011;32: 2125–2134. doi: 10.1093/eurheartj/ehr213 21697170

[26] Thuijs DJFM, Kappetein AP, Serruys PW, Mohr F-W, Morice M-C, Mack MJ, et al. Percutaneous coronary intervention versus coronary artery bypass grafting in patients with three-vessel or left main coronary artery disease: 10-year follow-up of the multicentre randomised controlled SYNTAX trial. Lancet. 2019;394: 1325–1334. doi: 10.1016/S0140-6736(19)31997-X 31488373

[27] Mehaffey JH, Hayanga JWA, Kawsara M, Sakhuja A, Mascio C, Rankin JS, et al. Contemporary coronary artery bypass grafting versus multivessel percutaneous coronary intervention. Ann Thorac Surg. 2023. doi: 10.1016/j.athoracsur.2023.05.032 37353103 PMC10739562

[28] Caldonazo T, Kirov H, Riedel LL, Gaudino M, Doenst T. Comparing CABG and PCI across the globe based on current regional registry evidence. Sci Rep-uk. 2022;12: 22164. doi: 10.1038/s41598-022-25853-4 36550130 PMC9780238

[29] Urso S, Sadaba R, Martín JMG, Dayan V, Nogales E, Tena MÁ, et al. Coronary surgery provides better survival than drug eluting stent: a pooled meta-analysis of Kaplan–Meier-derived individual patient data. J Thorac Cardiovasc Surg. 2023. doi: 10.1016/j.jtcvs.2023.03.020 37001801

[30] Sobolev BG, Levy AR, Kuramoto L, Hayden R, Brophy JM, Fitzgerald JM. The risk of death associated with delayed coronary artery bypass surgery. BMC health services research. 2006;6: 85. doi: 10.1186/1472-6963-6-85 16822309 PMC1574305

[31] Sobolev BG, Fradet G, Kuramoto L, Rogula B. The occurrence of adverse events in relation to time after registration for coronary artery bypass surgery: a population-based observational study. Journal of cardiothoracic surgery. 2013;8: 74. doi: 10.1186/1749-8090-8-74 23577641 PMC3639061

[32] VanderWeele TJ, Ding P. Sensitivity Analysis in Observational Research: Introducing the E-Value. Ann Intern Med. 2017;167: 268–274. doi: 10.7326/M16-2607 28693043

[33] Cochran WG, Chambers SP. The planning of observational studies of human populations. Journal of the Royal Statistical Society Series A (General). 1965;128: 234. doi: 10.2307/2344179

[34] Rosenbaum PR. Design of observational studies. Springer; 2010.

[35] Patel MR, Calhoon JH, Dehmer GJ, Grantham JA, Maddox TM, Maron DJ, et al. ACC/AATS/AHA/ASE/ASNC/SCAI/SCCT/STS 2017 appropriate use criteria for coronary revascularization in patients with stable ischemic heart disease: A report of the American College of Cardiology Appropriate Use Criteria Task Force, American Association for Thoracic Surgery, American Heart Association, American Society of Echocardiography, American Society of Nuclear Cardiology, Society for Cardiovascular Angiography and Interventions, Society of Cardiovascular Computed Tomography, and Society of Thoracic Surgeons. Journal of the American College of Cardiology. 2017;69: 2212–2241. doi: 10.1016/j.jacc.2017.02.001 28291663

[36] Sobolev B, Levy A, Hayden R, Kuramoto L. Does wait-list size at registration influence time to surgery? Analysis of a population-based cardiac surgery registry. Health services research. 2006;41: 23–39. doi: 10.1111/j.1475-6773.2005.00459.x 16430599 PMC1681524

