# Supplementary material for: The effect of coronary revascularization treatment timing on mortality in patients with stable ischemic heart disease in British Columbia
Source: PLoS One. 2024 Oct 24;19(10):e0303222. doi: 10.1371/journal.pone.0303222 (PMC11500866; doi:10.1371/journal.pone.0303222)
Supplement: S1 Appendix — (DOCX) [file pone.0303222.s001.docx]

**Mortality & Coronary Revascularization Treatment Timing**

**S1 Appendix**

***Part 1. Results of Analysis by Time Period***

In the first part of the supplementary material file, we provide the results of the mortality analysis by time period.

1. Mortality Analysis Results: 2001 – 2005
2. Mortality Analysis Results: 2006 – 2010
3. Mortality Analysis Results: 2011 – 2016

***Part 2. Supporting Information***

In the second part supplementary material file, we provide information on concept operationalization used in this study, as well as the STROBE Checklist.

1. Propensity Scores and Standardized Differences for Delayed Coronary Artery Bypass Grafting (CABG) in the Percutaneous Coronary Intervention (PCI) and CABG Populations
2. Wait Time Distribution by Study Group
3. Clearance Time Operationalization
4. Comorbidity Operationalization
5. Staged PCI Identification Algorithm
6. Repeat Revascularization Algorithm
7. STROBE Checklist for Cohort Studies
8. CSBC Data Set Background
9. Data Source Citations
10. References

***Part 1. Results of Mortality Analysis by Time Period***

**S1-1. Mortality Analysis Results: 2001 – 2005**

Figure S1-1-1. Standardized differences between study groups in propensity score model factors before and after inverse probability of treatment weighting, 2001 – 2005.

**
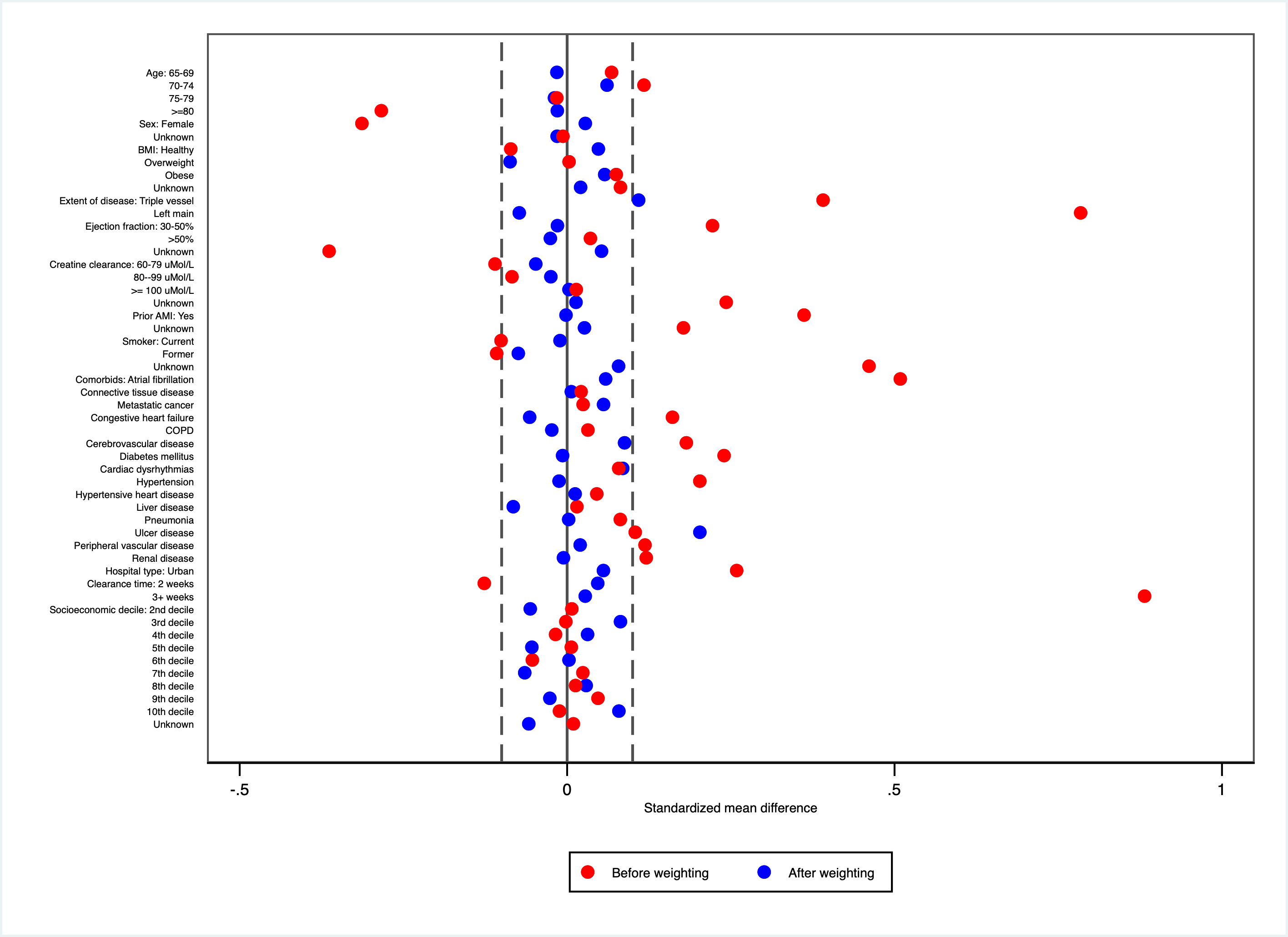
**

Table S1-1-1. Baseline characteristics of the patients, 2001 – 2005.

|  | **Unadjusted Data** | | | | | **Data Adjusted with**  **Inverse Probability Weighting** | | | | |
| --- | --- | --- | --- | --- | --- | --- | --- | --- | --- | --- |
|  | Timely PCI  (n= 5,650) | | Delayed CABG  (n= 2,929) | | P-Value | Timely PCI  (n= 8,932) | | Delayed CABG  (n= 7,794) | | P-Value |
|  | N | % | N | % |  | N | % | N | % |  |
| Age* |  |  |  |  |  |  |  |  |  |  |
| 60 - 64 | 1,131 | 20.0% | 645 | 22.0% | <0.00 | 1,790 | 20.0% | 1,503 | 19.3% | 0.90 |
| 65 - 69 | 1,205 | 21.3% | 708 | 24.2% |  | 1,903 | 21.3% | 1,611 | 20.7% |  |
| 70 - 74 | 1,225 | 21.7% | 782 | 26.7% |  | 2,210 | 24.7% | 2,137 | 27.4% |  |
| 75 - 79 | 1,131 | 20.0% | 568 | 19.4% |  | 1,761 | 19.7% | 1,477 | 18.9% |  |
| >=80 | 958 | 17.0% | 226 | 7.7% |  | 1,268 | 14.2% | 1,066 | 13.7% |  |
| Sex |  |  |  |  |  |  |  |  |  |  |
| Male | 3,847 | 68.1% | 2,388 | 81.5% | <0.00 | 6,534 | 73.1% | 5,607 | 71.9% | 0.69 |
| Female | 1,803 | 31.9% | 541 | 18.5% |  | 2,399 | 26.9% | 2,186 | 28.1% |  |
| Body Mass Index* |  |  |  |  |  |  |  |  |  |  |
| <18.5 | 67 | 1.2% | 15 | 0.5% | <0.00 | 87 | 1.0% | 30 | 0.4% | 0.28 |
| ≥18.5 and <25 | 1,589 | 28.1% | 713 | 24.3% |  | 2,426 | 27.2% | 2,284 | 29.3% |  |
| ≥25 and <30 | 2,538 | 44.9% | 1,320 | 45.1% |  | 4,121 | 46.1% | 3,259 | 41.8% |  |
| >30 | 1,397 | 24.7% | 821 | 28.0% |  | 2,174 | 24.3% | 2,092 | 26.8% |  |
| Missing | 59 | 1.0% | 60 | 2.0% |  | 125 | 1.4% | 129 | 1.6% |  |
| Extent of Disease |  |  |  |  |  |  |  |  |  |  |
| Double Vessel Disease | 3,039 | 53.8% | 165 | 5.6% | <0.00 | 3,194 | 35.8% | 2,584 | 33.2% | 0.24 |
| Triple Vessel Disease | 2,323 | 41.1% | 1,766 | 60.3% |  | 3,947 | 44.2% | 3,868 | 49.6% |  |
| Left Main Disease | 288 | 5.1% | 998 | 34.1% |  | 1,792 | 20.1% | 1,342 | 17.2% |  |
| Ejection Fraction† |  |  |  |  |  |  |  |  |  |  |
| EF <30% | 188 | 3.3% | 108 | 3.7% |  | 337 | 3.8% | 296 | 3.8% | 0.82 |
| EF ≥30% and ≤50% | 1,190 | 21.1% | 900 | 30.7% |  | 2,132 | 23.9% | 1,812 | 23.2% |  |
| EF >50% | 3,240 | 57.3% | 1,731 | 59.1% |  | 5,214 | 58.4% | 4,451 | 57.1% |  |
| Missing | 1,032 | 18.3% | 190 | 6.5% |  | 1,249 | 14.0% | 1,235 | 15.8% |  |
| Serum Creatinine (μmol/L)* |  |  |  |  |  |  |  |  |  |  |
| <60 | 120 | 2.1% | 47 | 1.6% | <0.00 | 169 | 1.9% | 334 | 4.3% | 0.38 |
| 60≥ and <80 | 1,038 | 18.4% | 419 | 14.3% |  | 1,635 | 18.3% | 1,285 | 16.5% |  |
| 80≥ and <99 | 1,977 | 35.0% | 909 | 31.0% |  | 2,973 | 33.3% | 2,503 | 32.1% |  |
| ≥100 | 2,053 | 36.3% | 1,084 | 37.0% |  | 3,093 | 34.6% | 2,709 | 34.8% |  |
|  |  |  |  |  |  | 1,063 | 11.9% | 962 | 12.3% |  |
| Prior Acute Myocardial Infarction* |  |  |  |  |  |  |  |  |  |  |
| Yes | 1,576 | 27.9% | 1,319 | 45.0% | <0.00 | 3,116 | 34.9% | 2,712 | 34.8% | 0.91 |
| No | 3,850 | 68.1% | 1,370 | 46.8% |  | 5,237 | 58.6% | 4,523 | 58.0% |  |
| Unknown | 224 | 4.0% | 240 | 8.2% |  | 580 | 6.5% | 558 | 7.2% |  |
| Smoking Status* |  |  |  |  |  |  |  |  |  |  |
| Never | 1,977 | 35.0% | 795 | 27.1% | <0.00 | 1,977 | 35.0% | 795 | 27.1% | 0.50 |
| Current/Now | 649 | 11.5% | 248 | 8.5% |  | 649 | 11.5% | 248 | 8.5% |  |
| Former/Quit | 2,614 | 46.3% | 1,199 | 40.9% |  | 2,614 | 46.3% | 1,199 | 40.9% |  |
| Unknown | 410 | 7.3% | 687 | 23.5% |  | 410 | 7.3% | 687 | 23.5% |  |
| Comorbidities |  |  |  |  |  |  |  |  |  |  |
| Atrial Fibrillation or Atrial Flutter | 432 | 7.6% | 765 | 26.1% | <0.00 | 1,189 | 13.3% | 1,198 | 15.4% | 0.32 |
| Cardiac Dysrhythmias§ | 286 | 5.1% | 203 | 6.9% | <0.00 | 522 | 5.8% | 623 | 8.0% | 0.17 |
| Cerebrovascular Disease | 138 | 2.4% | 179 | 6.1% | <0.00 | 387 | 4.3% | 491 | 6.3% | 0.40 |
| Chronic Pulmonary Disease | 309 | 5.5% | 182 | 6.2% | 0.16 | 503 | 5.6% | 398 | 5.1% | 0.60 |
| Congestive Heart Failure | 664 | 11.8% | 510 | 17.4% | <0.00 | 1,315 | 14.7% | 994 | 12.8% | 0.21 |
| Connective Tissue Disease | 85 | 1.5% | 52 | 1.8% | 0.34 | 125 | 1.4% | 115 | 1.5% | 0.87 |
| Diabetes | 1,341 | 23.7% | 1,012 | 34.6% | <0.00 | 2,529 | 28.3% | 2,182 | 28.0% | 0.91 |
| Hypertension | 1,925 | 34.1% | 1,286 | 43.9% | <0.00 | 3,181 | 35.6% | 2,730 | 35.0% | 0.84 |
| Hypertensive Heart Disease | 12 | 0.2% | 14 | 0.5% | 0.03 | 18 | 0.2% | 20 | 0.3% | 0.56 |
| Liver Disease | 13 | 0.2% | 9 | 0.3% | 0.50 | 84 | 0.9% | 23 | 0.3% | 0.16 |
| Metastatic Cancer | 138 | 2.4% | 83 | 2.8% | 0.28 | 226 | 2.5% | 271 | 3.5% | 0.40 |
| Peripheral Vascular Disease | 303 | 5.4% | 245 | 8.4% | <0.00 | 523 | 5.9% | 493 | 6.3% | 0.60 |
| Pneumonia | 141 | 2.5% | 115 | 3.9% | <0.00 | 269 | 3.0% | 238 | 3.1% | 0.95 |
| Renal Disease | 340 | 6.0% | 270 | 9.2% | <0.00 | 753 | 8.4% | 645 | 8.3% | 0.95 |
| Ulcer Disease | 35 | 0.6% | 51 | 1.7% | <0.00 | 63 | 0.7% | 284 | 3.6% | 0.00 |
| Hospital Type |  |  |  |  |  |  |  |  |  |  |
| Metropolitan | 4,801 | 85.0% | 2,187 | 74.7% | <0.00 | 7,089 | 79.4% | 6,007 | 77.1% | 0.43 |
| Urban | 849 | 15.0% | 742 | 25.3% |  | 1,843 | 20.6% | 1,787 | 22.9% |  |
| Clearance Time Category¶ |  |  |  |  |  |  |  |  |  |  |
| 1 Week | 3,543 | 62.7% | 849 | 29.0% | <0.00 | 4,380 | 49.0% | 3,573 | 45.8% | 0.53 |
| 2 Weeks | 1,188 | 21.0% | 472 | 16.1% |  | 1,782 | 19.9% | 1,703 | 21.8% |  |
| 3 or More Weeks | 919 | 16.3% | 1,608 | 54.9% |  | 2,771 | 31.0% | 2,518 | 32.3% |  |
| Neighborhood Income Decile |  |  |  |  |  |  |  |  |  |  |
| Lowest Decile | 592 | 10.5% | 292 | 10.0% | 0.31 | 915 | 10.2% | 783 | 10.0% | 0.62 |
| 2nd Decile | 597 | 10.6% | 316 | 10.8% |  | 1,021 | 11.4% | 756 | 9.7% |  |
| 3rd Decile | 582 | 10.3% | 300 | 10.2% |  | 877 | 9.8% | 964 | 12.4% |  |
| 4th Decile | 558 | 9.9% | 274 | 9.4% |  | 828 | 9.3% | 794 | 10.2% |  |
| 5th Decile | 541 | 9.6% | 286 | 9.8% |  | 802 | 9.0% | 584 | 7.5% |  |
| 6th Decile | 584 | 10.3% | 257 | 8.8% |  | 913 | 10.2% | 804 | 10.3% |  |
| 7th Decile | 512 | 9.1% | 286 | 9.8% |  | 895 | 10.0% | 636 | 8.2% |  |
| 8th Decile | 536 | 9.5% | 289 | 9.9% |  | 862 | 9.6% | 820 | 10.5% |  |
| 9th Decile | 477 | 8.4% | 287 | 9.8% |  | 754 | 8.4% | 602 | 7.7% |  |
| Highest Decile | 546 | 9.7% | 273 | 9.3% |  | 824 | 9.2% | 907 | 11.6% |  |
| Unknown | 125 | 2.2% | 69 | 2.4% |  | 243 | 2.7% | 143 | 1.8% |  |

* At the time of revascularization.

† Ejection Fraction at the time of revascularization; if missing, at the time of diagnostic catheterization.

§ Excluding atrial fibrillation and atrial flutter.

¶ Clearance time is the hypothetical time within which the wait list would be cleared at maximum weekly service capacity if there were no new arrivals.

Figure S1-1-2. Cumulative mortality in the CABG and PCI populations, 2001 – 2005, from an unadjusted analysis.

Table S1-1-2. Rates of mortality (percent), risk ratios, and 95% confidence intervals in the delayed CABG and timely PCI populations, 2001 – 2005 from an unadjusted analysis.

|  | 30 Days | 1 Year | 2 Years | 3 Years |
| --- | --- | --- | --- | --- |
| Delayed CABG | 1.0 (0.7, 1.4) | 3.1 (2.6, 3.7) | 4.8 (4.1, 5.5) | 6.5 (5.6, 7.4) |
| Timely PCI | 1.4 (1.2, 1.7) | 5.6 (5.0, 6.2) | 8.6 (7.9, 9.3) | 11.7 (10.9, 12.5) |
| Risk Ratio for Delayed CABG | 0.72 (0.45, 0.99) | 0.56 (0.45, 0.68) | 0.55 (0.46, 0.65) | 0.55 (0.47, 0.64) |

Table S1-1-3. Rates of mortality (percent), risk ratios, and 95% confidence intervals in the delayed CABG and timely PCI populations, 2001 – 2005, from an adjusted analysis.

|  | 30 Days | 1 Year | 2 Years | 3 Years |
| --- | --- | --- | --- | --- |
| Delayed CABG | 0.9 (0.4, 1.3) | 2.2. (1.5, 2.8) | 3.4 (2.6, 4.1) | 4.7 (3.7, 5.7) |
| Timely PCI | 2.3 (1.0, 3.6) | 7.6 (4.7, 10.6) | 10.9 (8.0 13.8) | 14.2 (11.2, 17.2) |
| Risk Ratio for Delayed CABG | 0.36 (0.12, 0.63) | 0.28 (0.14, 0.43) | 0.31 (0.19, 0.42) | 0.33 (0.23, 0.43) |

**S1-2. Mortality Analysis Results: 2006 – 2010**

Figure S1-2-1. Standardized differences between study groups in propensity score model factors before and after inverse probability of treatment weighting, 2006 – 2010.


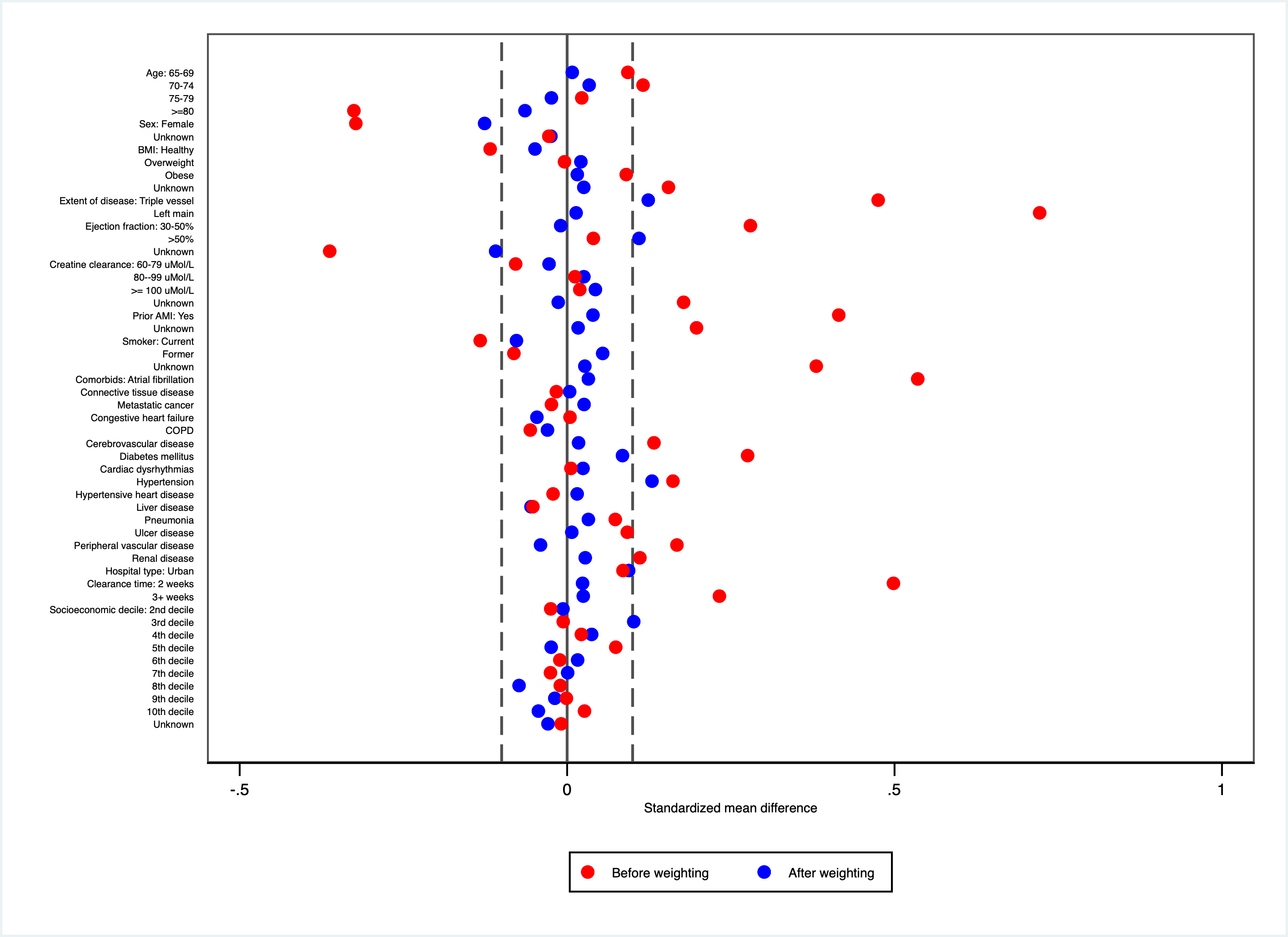


Table S1-2-1. Baseline characteristics of the patients, 2006 – 2010.

|  | **Unadjusted Data** | | | | | **Data Adjusted with**  **Inverse Probability Weighting** | | | | |
| --- | --- | --- | --- | --- | --- | --- | --- | --- | --- | --- |
|  | Timely PCI  (n= 7,672) | | Delayed CABG  (n= 2,221) | | P-Value | Timely PCI  (n= 9,786) | | Delayed CABG  (n= 8,685) | | P-Value |
|  | N | % | N | % |  | N | % | N | % |  |
| Age* |  |  |  |  |  |  |  |  |  |  |
| 60 - 64 | 1,575 | 20.5% | 477 | 22.5% | <0.00 | 2,008 | 20.5% | 1,927 | 22.2% | 0.81 |
| 65 - 69 | 1,594 | 20.8% | 523 | 24.7% |  | 2,093 | 21.4% | 1,886 | 21.7% |  |
| 70 - 74 | 1,479 | 19.3% | 510 | 24.0% |  | 2,028 | 20.7% | 1,920 | 22.1% |  |
| 75 - 79 | 1,430 | 18.6% | 414 | 19.5% |  | 1,807 | 18.5% | 1,524 | 17.5% |  |
| >=80 | 1,594 | 20.8% | 197 | 9.3% |  | 1,850 | 18.9% | 1,428 | 16.4% |  |
| Sex |  |  |  |  |  |  |  |  |  |  |
| Male | 5,362 | 69.9% | 1,769 | 83.4% | <0.00 | 7,062 | 72.2% | 6,744 | 77.6% | 0.08 |
| Female | 2,310 | 30.1% | 352 | 16.6% |  | 2,724 | 27.8% | 1,941 | 22.4% |  |
| Body Mass Index* |  |  |  |  |  |  |  |  |  |  |
| <18.5 | 95 | 1.2% | 18 | 0.8% | <0.00 | 115 | 1.2% | 126 | 1.4% | 0.87 |
| ≥18.5 and <25 | 2,312 | 30.1% | 528 | 24.9% |  | 2,900 | 29.6% | 2,381 | 27.4% |  |
| ≥25 and <30 | 3,296 | 43.0% | 907 | 42.8% |  | 4,183 | 42.7% | 3,802 | 43.8% |  |
| >30 | 1,931 | 25.2% | 619 | 29.2% |  | 2,529 | 25.8% | 2,304 | 26.5% |  |
| Missing | 38 | 0.5% | 49 | 2.3% |  | 59 | 0.6% | 71 | 0.8% |  |
| Extent of Disease |  |  |  |  |  |  |  |  |  |  |
| Double Vessel Disease | 4,165 | 54.3% | 129 | 6.1% | <0.00 | 4,293 | 43.9% | 3,236 | 37.3% | 0.10 |
| Triple Vessel Disease | 3,212 | 41.9% | 1,377 | 64.9% |  | 4,493 | 45.9% | 4,524 | 52.1% |  |
| Left Main Disease | 295 | 3.8% | 615 | 29.0% |  | 1,001 | 10.2% | 924 | 10.6% |  |
| Ejection Fraction† |  |  |  |  |  |  |  |  |  |  |
| EF <30% | 269 | 3.5% | 60 | 2.8% | <0.00 | 368 | 3.8% | 253 | 2.9% | 0.12 |
| EF ≥30% and ≤50% | 1,270 | 16.6% | 596 | 28.1% |  | 1,803 | 18.4% | 1,567 | 18.0% |  |
| EF >50% | 4,468 | 58.2% | 1,277 | 60.2% |  | 5,738 | 58.6% | 5,556 | 64.0% |  |
| Missing | 1,665 | 21.7% | 188 | 8.9% |  | 1,876 | 19.2% | 1,309 | 15.1% |  |
| Serum Creatinine (μmol/L)* |  |  |  |  |  |  |  |  |  |  |
| <60 | 332 | 4.3% | 59 | 2.8% | <0.00 | 386 | 3.9% | 181 | 2.1% | 0.26 |
| 60≥ and <80 | 1,953 | 25.5% | 469 | 22.1% |  | 2,449 | 25.0% | 2,070 | 23.8% |  |
| 80≥ and <99 | 2,781 | 36.2% | 781 | 36.8% |  | 3,578 | 36.6% | 3,283 | 37.8% |  |
| ≥100 | 2,441 | 31.8% | 694 | 32.7% |  | 3,102 | 31.7% | 2,929 | 33.7% |  |
|  | 165 | 2.2% | 118 | 5.6% |  | 271 | 2.8% | 221 | 2.5% |  |
| Prior Acute Myocardial Infarction* |  |  |  |  |  |  |  |  |  |  |
| Yes | 1,628 | 21.2% | 847 | 39.9% | <0.00 | 2,494 | 25.5% | 2,364 | 27.2% | 0.68 |
| No | 5,943 | 77.5% | 1,175 | 55.4% |  | 7,060 | 72.1% | 6,091 | 70.1% |  |
| Unknown | 101 | 1.3% | 99 | 4.7% |  | 232 | 2.4% | 229 | 2.6% |  |
| Smoking Status* |  |  |  |  |  |  |  |  |  |  |
| Never | 3,116 | 40.6% | 841 | 39.7% |  | 3,943 | 40.3% | 3,427 | 39.5% | 0.47 |
| Current/Now | 980 | 12.8% | 184 | 8.7% |  | 1,160 | 11.9% | 822 | 9.5% |  |
| Former/Quit | 3,441 | 44.9% | 866 | 40.8% |  | 4,356 | 44.5% | 4,101 | 47.2% |  |
| Unknown | 135 | 1.8% | 230 | 10.8% |  | 328 | 3.4% | 335 | 3.9% |  |
| Comorbidities |  |  |  |  |  |  |  |  |  |  |
| Atrial Fibrillation or Atrial Flutter | 578 | 7.5% | 575 | 27.1% | <0.00 | 1,068 | 10.9% | 1,038 | 11.9% | 0.40 |
| Cardiac Dysrhythmias§ | 281 | 3.7% | 80 | 3.8% | 0.81 | 358 | 3.7% | 359 | 4.1% | 0.65 |
| Cerebrovascular Disease | 170 | 2.2% | 98 | 4.6% | <0.00 | 264 | 2.7% | 259 | 3.0% | 0.73 |
| Chronic Pulmonary Disease | 365 | 4.8% | 77 | 3.6% | 0.03 | 433 | 4.4% | 333 | 3.8% | 0.47 |
| Congestive Heart Failure | 807 | 10.5% | 226 | 10.7% | 0.86 | 981 | 10.0% | 753 | 8.7% | 0.30 |
| Connective Tissue Disease | 89 | 1.2% | 21 | 1.0% | 0.51 | 121 | 1.2% | 111 | 1.3% | 0.93 |
| Diabetes | 2,108 | 27.5% | 857 | 40.4% | <0.00 | 2,945 | 30.1% | 2,955 | 34.0% | 0.16 |
| Hypertension | 3,833 | 50.0% | 1,230 | 58.0% | <0.00 | 5,177 | 52.9% | 5,152 | 59.3% | 0.05 |
| Hypertensive Heart Disease | 18 | 0.2% | NR | NR | 0.41 | 26 | 0.3% | 30 | 0.4% | 0.77 |
| Liver Disease | 19 | 0.2% | NR | NR | 0.07 | 19 | 0.2% | NR | NR | 0.00 |
| Metastatic Cancer | 202 | 2.6% | 48 | 2.3% | 0.34 | 245 | 2.5% | 254 | 2.9% | 0.56 |
| Peripheral Vascular Disease | 273 | 3.6% | 156 | 7.4% | <0.00 | 404 | 4.1% | 292 | 3.4% | 0.14 |
| Pneumonia | 221 | 2.9% | 90 | 4.2% | 0.00 | 314 | 3.2% | 330 | 3.8% | 0.48 |
| Renal Disease | 412 | 5.4% | 173 | 8.2% | <0.00 | 564 | 5.8% | 558 | 6.4% | 0.66 |
| Ulcer Disease | 33 | 0.4% | 27 | 1.3% | <0.00 | 56 | 0.6% | 54 | 0.6% | 0.79 |
| Hospital Type |  |  |  |  |  |  |  |  |  |  |
| Metropolitan | 6,452 | 84.1% | 1,715 | 80.9% | <0.00 | 8,246 | 84.3% | 7,007 | 80.7% | 0.23 |
| Urban | 1,220 | 15.9% | 406 | 19.1% |  | 1,541 | 15.7% | 1,677 | 19.3% |  |
| Clearance Time Category¶ |  |  |  |  |  |  |  |  |  |  |
| 1 Week | 6,242 | 81.4% | 1,153 | 54.4% | <0.00 | 7,350 | 75.1% | 6,385 | 73.5% | 0.67 |
| 2 Weeks | 901 | 11.7% | 672 | 31.7% |  | 1,614 | 16.5% | 1,509 | 17.4% |  |
| 3 or More Weeks | 529 | 6.9% | 296 | 14.0% |  | 822 | 8.4% | 790 | 9.1% |  |
| Neighborhood Income Decile |  |  |  |  |  |  |  |  |  |  |
| Lowest Decile | 852 | 11.1% | 207 | 9.8% | 0.11 | 1,066 | 10.9% | 958 | 11.0% | 0.82 |
| 2nd Decile | 818 | 10.7% | 210 | 9.9% |  | 1,014 | 10.4% | 884 | 10.2% |  |
| 3rd Decile | 755 | 9.8% | 205 | 9.7% |  | 935 | 9.6% | 1,108 | 12.8% |  |
| 4th Decile | 823 | 10.7% | 242 | 11.4% |  | 1,077 | 11.0% | 1,060 | 12.2% |  |
| 5th Decile | 755 | 9.8% | 258 | 12.2% |  | 1,032 | 10.5% | 852 | 9.8% |  |
| 6th Decile | 734 | 9.6% | 196 | 9.2% |  | 891 | 9.1% | 831 | 9.6% |  |
| 7th Decile | 714 | 9.3% | 182 | 8.6% |  | 933 | 9.5% | 830 | 9.6% |  |
| 8th Decile | 758 | 9.9% | 203 | 9.6% |  | 942 | 9.6% | 657 | 7.6% |  |
| 9th Decile | 715 | 9.3% | 197 | 9.3% |  | 922 | 9.4% | 771 | 8.9% |  |
| Highest Decile | 665 | 8.7% | 200 | 9.4% |  | 861 | 8.8% | 659 | 7.6% |  |
| Unknown | 83 | 1.1% | 21 | 1.0% |  | 113 | 1.2% | 75 | 0.9% |  |

* At the time of revascularization.

† Ejection Fraction at the time of revascularization; if missing, at the time of diagnostic catheterization.

§ Excluding atrial fibrillation and atrial flutter.

¶ Clearance time is the hypothetical time within which the wait list would be cleared at maximum weekly service capacity if there were no new arrivals.

Abbreviations: NR, not reported due to small cell size.

Figure S1-2-2. Cumulative mortality in the CABG and PCI populations, 2006 – 2010, from an unadjusted analysis.

Table S1-2-2. Rates of mortality (percent), risk ratios, and 95% confidence intervals in the delayed CABG and timely PCI populations, 2006 – 2010, from an unadjusted analysis, 2006 – 2010.

|  | 30 Days | 1 Year | 2 Years | 3 Years |
| --- | --- | --- | --- | --- |
| Delayed CABG | 0.5 (0.3, 0.8) | 2.3 (1.7, 2.9) | 3.9 (3.2, 4.7). | 5.7 (4.7, 6.6) |
| Timely PCI | 1.5 (1.3, 1.7) | 5.6 (5.1, 6.1) | 8.6 (8.0, 9.2) | 11.7 (11.0, 12.5) |
| Risk Ratio for Delayed CABG | 0.36 (0.17, 0.54) | 0.42 (0.31, 0.53) | 0.46 (0.36, 0.55) | 0.48 (0.39, 0.57) |

Table S1-2-3. Rates of mortality (percent), risk ratios, and 95% confidence intervals in the delayed CABG and timely PCI populations, 2006 – 2010, from an adjusted analysis.

|  | 30 Days | 1 Year | 2 Years | 3 Years |
| --- | --- | --- | --- | --- |
| Delayed CABG | 2.4 (-1.4, 6.1) | 4.4 (0.00, 9.3) | 5.2 (2.0, 10.3) | 6.2 (0.8, 11.7) |
| Timely PCI | 1.7 (1.4, 2.0) | 5.3 (4.7, 5.9) | 8.3 (7.6, 9.1) | 11.7 (10.8, 12.6) |
| Risk Ratio for Delayed CABG | 1.39 (-0.87, 3.65) | 0.82 (-0.13, 1.77) | 0.63 (0.9, 1.25) | 0.53 (0.062, 1.01) |

**S1-3. Mortality Analysis: 2011 – 2016**

Figure S1-3-1. Standardized differences between study groups in propensity score model factors before and after inverse probability of treatment weighting, 2011– 2016.


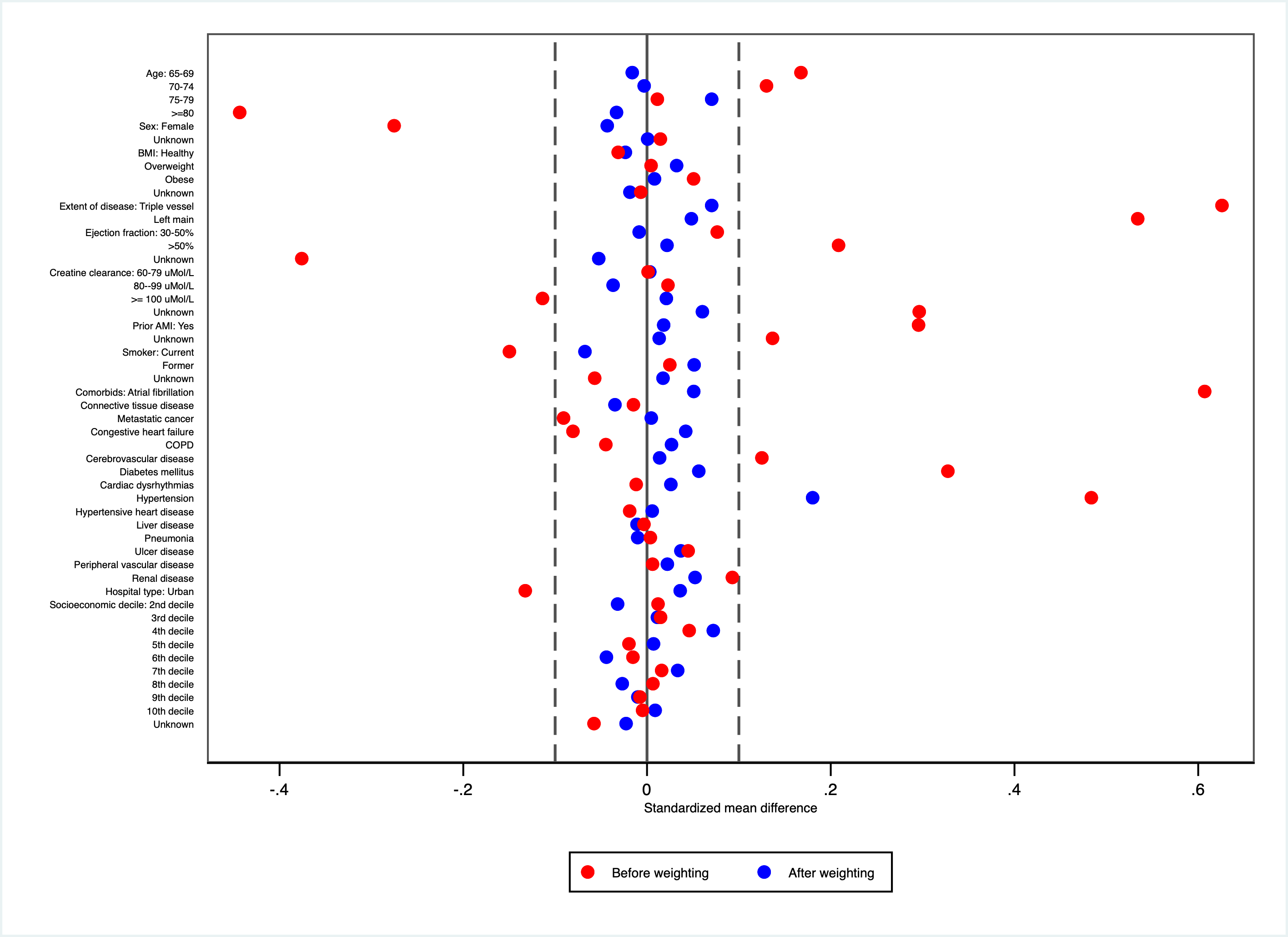


Table S1-3-1. Baseline characteristics of the patients, 2011– 2016.

|  | **Unadjusted Data** | | | | | **Data Adjusted with**  **Inverse Probability Weighting** | | | | |
| --- | --- | --- | --- | --- | --- | --- | --- | --- | --- | --- |
|  | Timely PCI  (n=6,248) | | Delayed CABG  (n= 2,475) | | P-Value | Timely PCI  (n= 8,611) | | Delayed CABG  (n= 8,233) | | P-Value |
|  | N | % | N | % |  | N | % | N | % |  |
| Age* |  |  |  |  |  |  |  |  |  |  |
| 60 - 64 | 1,168 | 18.7% | 542 | 21.9% | <0.00 | 1,654 | 19.2% | 1,531 | 18.6% | 0.67 |
| 65 - 69 | 1,299 | 20.8% | 692 | 28.0% |  | 1,942 | 22.6% | 1,802 | 21.9% |  |
| 70 - 74 | 1,181 | 18.9% | 600 | 24.2% |  | 1,785 | 20.7% | 1,696 | 20.6% |  |
| 75 - 79 | 1,054 | 16.9% | 428 | 17.3% |  | 1,475 | 17.1% | 1,635 | 19.9% |  |
| >=80 | 1,546 | 24.7% | 213 | 8.6% |  | 1,755 | 20.4% | 1,569 | 19.1% |  |
| Sex |  |  |  |  |  |  |  |  |  |  |
| Male | 4,434 | 71.0% | 2,041 | 82.5% | <0.00 | 6,377 | 74.0% | 6,251 | 75.9% | 0.47 |
| Female | 1,814 | 29.0% | 434 | 17.5% |  | 2,235 | 26.0% | 1,982 | 24.1% |  |
| Body Mass Index* |  |  |  |  |  |  |  |  |  |  |
| <18.5 | 90 | 1.4% | 11 | 0.4% | <0.00 | 99 | 1.1% | 29 | 0.3% | 0.20 |
| ≥18.5 and <25 | 1,892 | 30.3% | 714 | 28.8% |  | 2,517 | 29.2% | 2,319 | 28.2% |  |
| ≥25 and <30 | 2,632 | 42.1% | 1,048 | 42.3% |  | 3,689 | 42.8% | 3,659 | 44.4% |  |
| >30 | 1,614 | 25.8% | 695 | 28.1% |  | 2,282 | 26.5% | 2,212 | 26.9% |  |
| Missing | 20 | 0.3% | 7 | 0.3% |  | 24 | 0.3% | 15 | 0.2% |  |
| Extent of Disease |  |  |  |  |  |  |  |  |  |  |
| Double Vessel Disease | 3,676 | 58.8% | 265 | 10.7% | <0.00 | 3,935 | 45.7% | 3,349 | 40.7% | 0.09 |
| Triple Vessel Disease | 2,226 | 35.6% | 1,621 | 65.5% |  | 3,808 | 44.2% | 3,929 | 47.7% |  |
| Left Main Disease | 346 | 5.5% | 589 | 23.8% |  | 870 | 10.1% | 955 | 11.6% |  |
| Ejection Fraction† |  |  |  |  |  |  |  |  |  |  |
| EF <30% | 304 | 4.9% | 107 | 4.3% | <0.00 | 455 | 5.3% | 531 | 6.5% | 0.62 |
| EF ≥30% and ≤50% | 1,188 | 19.0% | 547 | 22.1% |  | 1,786 | 20.7% | 1,679 | 20.4% |  |
| EF >50% | 3,514 | 56.2% | 1,642 | 66.3% |  | 4,960 | 57.6% | 4,830 | 58.7% |  |
| Missing | 1,242 | 19.9% | 179 | 7.2% |  | 1,411 | 16.4% | 1,192 | 14.5% |  |
| Serum Creatinine (μmol/L)* |  |  |  |  |  |  |  |  |  |  |
| <60 | 406 | 6.5% | 125 | 5.1% | <0.00 | 521 | 6.1% | 484 | 5.9% | 0.65 |
| 60≥ and <80 | 1,779 | 28.5% | 706 | 28.5% |  | 2,456 | 28.5% | 2,359 | 28.6% |  |
| 80≥ and <99 | 2,136 | 34.2% | 873 | 35.3% |  | 2,977 | 34.6% | 2,702 | 32.8% |  |
| ≥100 | 1,877 | 30.0% | 618 | 25.0% |  | 2,530 | 29.4% | 2,498 | 30.3% |  |
|  | 50 | 0.8% | 153 | 6.2% |  | 129 | 1.5% | 191 | 2.3% |  |
| Prior Acute Myocardial Infarction* |  |  |  |  |  |  |  |  |  |  |
| Yes | 528 | 8.5% | 456 | 18.4% | <0.00 | 1,038 | 12.0% | 1,041 | 12.6% | 0.79 |
| No | 1,648 | 26.4% | 249 | 10.1% |  | 1,882 | 21.9% | 1,698 | 20.6% |  |
| Unknown | 4,072 | 65.2% | 1,770 | 71.5% |  | 5,693 | 66.1% | 5,494 | 66.7% |  |
| Smoking Status* |  |  |  |  |  |  |  |  |  |  |
| Never | 2,542 | 40.7% | 1,108 | 44.8% | <0.00 | 3,611 | 41.9% | 3,395 | 41.2% | 0.51 |
| Current/Now | 829 | 13.3% | 213 | 8.6% |  | 1,011 | 11.7% | 795 | 9.7% |  |
| Former/Quit | 2,773 | 44.4% | 1,129 | 45.6% |  | 3,862 | 44.8% | 3,902 | 47.4% |  |
| Unknown | 104 | 1.7% | 25 | 1.0% |  | 128 | 1.5% | 141 | 1.7% |  |
| Comorbidities |  |  |  |  |  |  |  |  |  |  |
| Atrial Fibrillation or Atrial Flutter | 513 | 8.2% | 776 | 31.4% | <0.00 | 1,180 | 13.7% | 1,275 | 15.5% | <0.00 |
| Cardiac Dysrhythmias§ | 210 | 3.4% | 78 | 3.2% | 0.62 | 323 | 3.7% | 350 | 4.3% | 0.62 |
| Cerebrovascular Disease | 147 | 2.4% | 115 | 4.6% | <0.00 | 257 | 3.0% | 265 | 3.2% | 0.68 |
| Chronic Pulmonary Disease | 305 | 4.9% | 98 | 4.0% | 0.06 | 409 | 4.7% | 439 | 5.3% | 0.55 |
| Congestive Heart Failure | 791 | 12.7% | 250 | 10.1% | <0.00 | 1,119 | 13.0% | 1,189 | 14.4% | 0.40 |
| Connective Tissue Disease | 70 | 1.1% | 24 | 1.0% | 0.54 | 95 | 1.1% | 63 | 0.8% | 0.22 |
| Diabetes | 1,920 | 30.7% | 1,150 | 46.5% | <0.00 | 3,044 | 35.3% | 3,133 | 38.1% | 0.24 |
| Hypertension | 4,201 | 67.2% | 2,153 | 87.0% | <0.00 | 6,260 | 72.7% | 6,612 | 80.3% | <0.00 |
| Hypertensive Heart Disease | 9 | 0.1% | NR | NR | 0.45 | 11 | 0.1% | 12 | 0.1% | 0.87 |
| Liver Disease | 24 | 0.4% | 9 | 0.4% | 0.89 | 39 | 0.5% | 31 | 0.4% | 0.75 |
| Metastatic Cancer | 196 | 3.1% | 43 | 1.7% | <0.00 | 234 | 2.7% | 230 | 2.8% | 0.94 |
| Peripheral Vascular Disease | 178 | 2.8% | 73 | 2.9% | 0.80 | 241 | 2.8% | 262 | 3.2% | 0.62 |
| Pneumonia | 213 | 3.4% | 86 | 3.5% | 0.88 | 311 | 3.6% | 282 | 3.4% | 0.83 |
| Renal Disease | 558 | 8.9% | 291 | 11.8% | <0.00 | 883 | 10.3% | 980 | 11.9% | 0.36 |
| Ulcer Disease | 44 | 0.7% | 28 | 1.1% | 0.05 | 70 | 0.8% | 97 | 1.2% | 0.49 |
| Hospital Type |  |  |  |  |  |  |  |  |  |  |
| Metropolitan | 3,833 | 61.3% | 1,675 | 67.7% | <0.00 | 5,448 | 63.3% | 5,064 | 61.5% | 0.49 |
| Urban | 2,415 | 38.7% | 800 | 32.3% |  | 3,164 | 36.7% | 3,169 | 38.5% |  |
| Neighborhood Income Decile |  |  |  |  |  |  |  |  |  |  |
| Lowest Decile | 655 | 10.5% | 234 | 9.5% | 0.25 | 873 | 10.1% | 772 | 9.4% | 0.89 |
| 2nd Decile | 646 | 10.3% | 265 | 10.7% |  | 875 | 10.2% | 758 | 9.2% |  |
| 3rd Decile | 616 | 9.9% | 255 | 10.3% |  | 842 | 9.8% | 833 | 10.1% |  |
| 4th Decile | 671 | 10.7% | 302 | 12.2% |  | 953 | 11.1% | 1,106 | 13.4% |  |
| 5th Decile | 622 | 10.0% | 232 | 9.4% |  | 830 | 9.6% | 810 | 9.8% |  |
| 6th Decile | 552 | 8.8% | 208 | 8.4% |  | 743 | 8.6% | 611 | 7.4% |  |
| 7th Decile | 599 | 9.6% | 249 | 10.1% |  | 833 | 9.7% | 880 | 10.7% |  |
| 8th Decile | 589 | 9.4% | 238 | 9.6% |  | 810 | 9.4% | 711 | 8.6% |  |
| 9th Decile | 633 | 10.1% | 245 | 9.9% |  | 917 | 10.6% | 852 | 10.3% |  |
| Highest Decile | 597 | 9.6% | 233 | 9.4% |  | 857 | 9.9% | 841 | 10.2% |  |
| Unknown | 68 | 1.1% | 14 | 0.6% |  | 79 | 0.9% | 59 | 0.7% |  |

* At the time of revascularization.

† Ejection Fraction at the time of revascularization; if missing, at the time of diagnostic catheterization.

§ Excluding atrial fibrillation and atrial flutter.

Abbreviations: NR, not reported due to small cell size.

Figure S1-3-2. Cumulative mortality in the CABG and PCI populations, 2011– 2016, from an unadjusted analysis.

Table S1-3-2. Rates of mortality (percent), risk ratios, and 95% confidence intervals in the delayed CABG and timely PCI populations, 2006 – 2010, from an unadjusted analysis, 2011– 2016.

|  | 30 Days | 1 Year | 2 Years | 3 Years |
| --- | --- | --- | --- | --- |
| Delayed CABG | 0.5 (0.2, 0.7) | 2.2 (1.7, 2.8) | 3.9 (3.1, 4.6) | 5.5 (4.5, 6.6) |
| Timely PCI | 1.7 (1.4, 2.0) | 6.3 (5.7, 6.9) | 9.8 (9.1, 10.6) | 13.2 (12.2, 14.2) |
| Risk Ratio for Delayed CABG | 0.27 (0.13, 0.41) | 0.35 (0.26, 0.46) | 0.39 (0.31, 0.47) | 0.42 (0.33, 0.51) |

Table S1-3-3. Rates of mortality (percent), risk ratios, and 95% confidence intervals in the delayed CABG and timely PCI populations, 2011– 2016from an adjusted analysis.

|  | 30 Days | 1 Year | 2 Years | 3 Years |
| --- | --- | --- | --- | --- |
| Delayed CABG | 1.9 (-1.3, 5.1) | 4.3 (0.5, 8.0) | 5.7 (1.7, 9.7) | 7.0 (2.5, 11.5) |
| Timely PCI | 1.8 (1.4, 2.1) | 6.3 (5.4, 7.3) | 10.0 (8.9, 11.1) | 13.7 (12.4, 15.0) |
| Risk Ratio for Delayed CABG | 1.09 (-0.72, 2.90) | 0.68 (0.03, 1.32) | 0.57 (0.14, 0.99) | 0.51 (0.18, 0.84) |

***Part 2. Supplementary Material***

**S2-1. Propensity Scores and Standardized Differences for Delayed Coronary Artery Bypass Grafting (CABG) in the Percutaneous Coronary Intervention (PCI) and CABG populations.**

Figure S2-1-1. Propensity scores for delayed Coronary Artery Bypass Grafting (CABG) in the Percutaneous Coronary Intervention (PCI) and CABG populations.


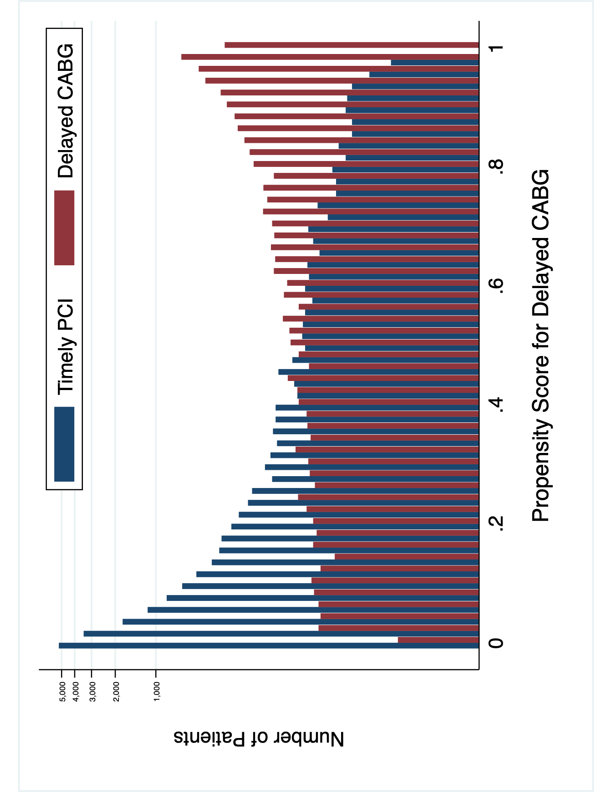


Figure S2-1-2. Standardized differences between study groups in propensity score model factors before and after inverse probability of treatment weighting.

**S2-2. Wait Time Distribution Median, Mean, and 90^th^ Percentile by Study Group**

Table S2-2-1.

Median, mean, and 90th percentile wait times by study group, in days.

| **Study Group** | **Mean** | **50^th^ Percentile** | **90^th^ Percentile** |
| --- | --- | --- | --- |
| Timely PCI | 11.9 days | 2 days | 30 days |
| Delayed CABG | 71.5 days | 45 days | 160 days |

**S2-3. Clearance Time Operationalization**

*Background*

Clearance time is the hypothetical time within which the list will be cleared at maximum capacity if there are no new arrivals.^1^

For each patient, we estimate the clearance time by dividing the number of pre-surgical or pre-catheterization patients present on the waiting list on the day a patient is booked for their procedure by the maximum weekly procedure rate at the same hospital.

We use the waiting list calculation proposed by Cottrell^2^ and stratify the waiting list into four strata as used by Sobolev et al.^1^ We categorize the list size as a function of clearance time. Clearance time is the hypothetical time within which the list will be cleared at maximum capacity if there are no new arrivals. We divide the list into four wait list categories: (1) where capacity is adequate to meet demand (2) where there is a short clearance time, or (3) where there is a prolonged clearance time. We will use weekly clearance time as the reference value.

Table S2-3-1.

Clearance Times Used to Estimating Demand for Coronary Revascularization Procedures

| **Category** | **Category Label** | **Clearance Time** |
| --- | --- | --- |
| 1 | 1 Week | <1 Week |
| 2 | 2 Weeks | ≥ 1 Week to  <2 Weeks |
| 3 | 3 or More Weeks | ≥ 2 Weeks |

Originally, we established four wait list categories characterizing clearance time, based on the recommendations made by the Canadian Cardiovascular Society’s Access to Care Working Group^3^ for access to cardiac surgery and cardiac catheterization services. For both procedures, access is recommended within two weeks for urgent patients and six weeks for all other patients. However, initial implementation suggested that a small number of CABG patients had a clearance time greater than six weeks, and no PCI patients had a clearance time of greater than six weeks. Therefore, the few CABG patients in the greater than six-week category were combined with the greater than two-week category to provide for meaningful categorizations.

*Clearance Time Calculation*

For each patient, we estimate the clearance time by dividing the number of pre-surgical or pre-catheterization patients present on the waiting list on the day a patient is booked for their procedure by the maximum weekly procedure rate at the same hospital.

We count the number of patients present on the waiting list based on (1) the existing list size at the time the patient is booked and (2) the batch size of new arrivals. ^1^ For each patient, the list size is a census of patients with equal or higher priority present at the date of booking in one of the cardiac centres. Patients contribute one count to the list size for each week on the list, except for the week of arrival. Patients who underwent revascularization are considered removed from the waitlist in the week prior to their procedure, as patients are scheduled on a weekly basis. For each patient, the batch size is the count of patients with higher or equal priority registered on the list in the same week.

The maximum rate estimates the largest number of cardiac surgeries or cardiac catheterizations a hospital can deliver during a week, thereby providing means to estimate the hospital’s capacity to manage existing demand.

**Clearance Time Dependencies**

Clearance time depends on several features of the revascularization system.

Table S2-3-2.

Maximum Weekly Procedure Rates for Cardiac Surgery and Cardiac Catheterization Lab Procedures at British Columbia Cardiac Centres

| **Cardiac Centre** | **Weekly Cardiac Surgery Procedures** | **Weekly Cardiac Catheterization Lab Procedures** |
| --- | --- | --- |
| Kelowna General Hospital | 12 | 60 |
| Royal Columbian Hospital | 20 | 88 |
| Royal Jubilee Hospital | 15 | 60 |
| St. Paul’s Hospital | 19 | 55 |
| Vancouver General Hospital | 16 | 75 |

Table S2-3-2 summarizes select features of the coronary revascularization system relevant to the estimation of clearance time. We obtained the weekly cardiac surgery and weekly cardiac catheterization lab procedure volumes from Cardiac Services BC (CSBC), who sought data from the triage coordinators for each site and service. These figures constitute site capacity in our operationalization of demand. We validated these figures as representative of the study period through personal communication with cardiac surgery division heads and site interventional cardiology medical directors for each site.

*Clearance Time by Revascularization Procedure*

CABG and PCI clearance times are different procedures, with different resource requirements. We account for these differences by separating the calculations for cardiac catheterization procedures and cardiac operating room procedures.

While the calculation of clearance time is for cardiac surgery requires only counting cardiac surgery cases, the cardiac catheterization lab procedures require counting both diagnostic catheterization cases and scheduled PCI cases as a procedure slot can be used for both procedures. We count diagnostic catheterization cases, ad-hoc PCI cases, and scheduled PCI cases in this category, as each consumes a cardiac catheterization procedure slot.

*Clearance Time and Booking Hospital*

CABG and PCI clearance times are dependent on hospital capacity. Therefore, we account for differences using capacity estimates obtained from the cardiac centres.

If a patient record has an unknown booking hospital, we use instead the hospital that performs the revascularization as the booking hospital. If a patient record has a booking hospital where only diagnostic catheterizations are performed, specifically Lion’s Gate Hospital, then we attribute demand to the revascularizing hospital.

*Clearance Time and Booking Priority*

CABG and PCI clearance times are dependent on booking priority. Therefore, we account for booking priority in the clearance time calculation.

If a patient record has an unknown priority at time of booking, we use the priority at the time of revascularization.

*Clearance Time and Calendar Week of Booking & Procedure*

CABG and PCI clearance times are dependent on the calendar week of the booking procedure. Therefore, we account for calendar week in the batch size calculation used in estimating clearance time.

If a patient has an unknown booking date, we use the date of diagnostic catheterization as the proxy booking date. If a patient has an unknown booking date for the diagnostic catheterization, we will assume that the booking happened the same week of the procedure.

**S2-4. Comorbidity Operationalization**

We used linked care episodes with hospitalization records in the Discharge Abstract Database to identify comorbidities. To identify comorbidities, we searched for diagnosis codes between the date of revascularization and the date that is one year prior to the date of the diagnostic catheterization.

Comorbidity codes were identified through previous work completed by members of the study team, and informed by clinical advisor feedback, and the Canadian Institute for Health Information (CIHI) and Canadian Cardiovascular Society’s (CCS) Cardiac Care Quality Indicators (CCQI) initiative methodology.

Table S2-4-1. ICD-10-CA and ICD-9 codes used to identify comorbidities.

| **Comorbid condition** | **ICD-10-CA code** | **ICD-9 code** |
| --- | --- | --- |
| Any Tumor or Metastatic Solid Tumor* | C00*-C75*;  C77*;  C78*;  C79*;  C80*; | 140 – 195;  196;  197;  198;  199; |
| Atrial Fibrillation or Atrial Flutter | I48.*; | 427.3; |
| Cardiac Dysrhythmias Excluding Atrial Fibrillation and Atrial Flutter | I47.*;  I49.* | 427.8, 427.0, 427.1, 427.2;  427.4, 427.6, 427.8, 427.9 |
| Cerebrovascular Disease | I60*;  I61*;  I62*;  I63*;  I64*;  I65*;  I67*;  I68*;  G45*;  G81*; | 430;  431;  432.1, 432.0, 432.9;  433.9, 434.0, 434.1, 434.9, 437.6;  436;  433.2, 433.0, 433.1, 433.3, 433.8. 433.9;  437.8, 437.3, 437.0, 437.2, 437.5, 437.6, 437.4, 437.9;  437.8, 437.4, 437.8;  435, 362.3;  342.0, 342.1, 342.9; |
| Chronic Pulmonary Disease | J40*;  J41*;  J42*;  J43*;  J44*;  J45*;  J47* | 490;  491.0, 491.1, 491.8;  491.9;  492;  496, 491.2;  493.0, 493.1, 493.9;  494; |
| Congestive Heart Failure | I50* | 428 |
| Connective Tissue Disease | M30*;    M31*,  M32*,    M33*,  M34*,  M35*,  M36*,  M05*,  M06*,  M07*,  M08*,  M09*,  M10*,  M11*,  M12*,  M13*,    M14* | 446.0, 446.4, 447.8, 446.1;  446.2, 446.6, 446.3, 446.4, 446.7. 446.5, 446.0, 447.8, 447.5;  710.0;  710.3, 710.4;  710.1;  710.2, 710.8, 136.1, 725, 729.4, 710.8, 729.3, 728.5, 710.8, 710.9;  710.3, 713.2, 713.6, 713.0;  714.1, 714.8, 714.2, 714.0;  714.0;  713.3, 713.1;  714.3, 720.0, 714.3;  714.3;  274.0. 984.9, 274.9, 274.8;  275.4, 712.8, 712.9;  714.4, 716.0, 719.2, 719.3, 716.1, 716.8;  716.5, 716.6, 716.8, 716.9;  713.0, 713.3, 713,7, 713.5, 713.8 |
| Dementia | F00*;  F01*;  F02*;  F03*;  F10*; | 290.1;  290.0;  290.8;  No value;  305.0, 303, 291.8, 291.3, 291.1, 291.2, 291.9; |
| Diabetes | E10*;  E11*;  E13*;  E14*; | 250.2, 250.1, 250.3, 250.4; 250.5, 250.6, 250.7, 250.0;  250.2, 250.1, 250.3, 250.4,  250.5, 250.6, 250.7, 250.0;  250.1, 250.3, 250.4, 250.5, 250.6, 250.7, 250.0; |
| Endocarditis | I01.1;  I33.*;    I38.*;  I39.8; | 391.1;  421.0, 421.9;  424.9;  424.9; |
| Hypertension | I10.*;  I12.*;  I13.*;  I15.*; | 401.1, 401.0;  403.9;  404.9;  405.9, 405.0; |
| Hypertensive heart disease, other rheumatic heart diseases | I09*,  I11* | 398.0, 397.9, 393, 398.9;  402.9; |
| Leukemia* | C91*;  C92*;  C93*;  C94*;  C95*;  C96* | 204.0, 204.1, 204.9, 202.4, 204.8, 204.9;  205.0, 205.1, 205.3, 205.8, 205.9;  206.0, 206.8, 206.9;  207.0, 207.2, 207.8, 289.8, 207.8;  208.0, 208.1, 208.8, 208.9;  202.5, 202.3, 202.6, 200.0, 202.8, 202.9; |
| Liver Disease (Mild or Moderate or Severe or Viral) | K70*;  K71*;  K72*;  K73*,  K74*,  K75*,  K76*,  K77*;  B15*;    B16*;  B17*;    B18*;  B19*; | 571.0, 571.1, 571.2, 572.8, 571.3;  573.3;  570, 572.8;  571.4;  571.5, 571.9, 571.6;  572.0, 572.1, 572.3;  571.8, 573.0, 570, 573.4, 573.8, 572.3, 572.4, 573.8, 573.9;  573.2, 572.8;  070.0, 070.1;  070.2, 070.3;  070.5, 070.9;  070.3. 070.5, 070.9;  070.6, 070.9; |
| Lymphoma* | C81*;  C82*;    C83*;  C84*;    C85*;  C86*;    C88*;    C90*; | 201.4, 201.5, 201.6, 201.7, 201.1, 201.9;  202.0, 202.8  202.8, 200.8, 200.1, 200.2;  202.1, 202.2, 202.0;  202.8;  No Code;  273.3, 200.8, 203.8;  203.0; |
| Peripheral Vascular Disease | I70;    I71*;  I72*;  I73*;  I74*;  I77*;  I78*;  I79*;  M30*; | 440.0, 440.1, 440.2, 440.8, 440.9;  441.0, 441.1, 441.2, 441.3, 441.4, 441.5, 441.5, 441.6;  442.8, 442.0, 442.1, 442.2, 442.3, 442.8, 442.9;  443.0, 443.1, 443.8, 443.9;  444.0, 444.1, 444.2, 444.8, 444.9;  447.0, 447.1, 447.2, 447.8, 447.4, 447.5, 447.6, 447.8, 447.9;  448.0, 448.1, 448.9;  441.7, 447.7, 443.8, 448.9;  446.0; |
| Pneumonia | J10.0;  J11.0;  J12.*;  J13;  J14;  J15.*;  J16.8;  J18.*;  J85.1; | 487.0;  487.0;  480.0, 480.1, 480.2, 480.8, 480.9;  481;  482.2;  482.0, 482.1, 482.4, 482.3, 482.8, 483, 482.9;  483;  485, 481, 514, 486;  513.0; |
| Renal Disease (Moderate or Severe) | I12*;  I13*;  N00*;  N01*;  N02*;  N03*;  N04*;  N05*;  N17*;  N18*;  N19*; | 403.9;  404.9;  580.8. 580.0, 580.9;  583.4;  599.7;  582.8, 582.1, 582.0, 582.2, 582.9;  581.3, 581.1, 581.0, 581.2, 581.8, 581.9;  581.3, 583.8, 583.1, 583.0, 583.2, 583.9;  584.5, 584.6, 584.7, 584.8, 584.9;  585;  586; |
| Ulcer Disease | K25*;  K26*;    K27*,  K28* | 531.0, 531.1, 531.2, 531.3, 531.4, 531.5, 531.6, 531.7, 531.9;  532.0, 532.1, 532.2, 532.3, 532.4, 532.5, 532.6, 532.7, 532.9;  533.0, 533.1, 533.2, 533.3, 533.4, 533.5, 533.5 533.6, 533.7, 533.9;  534.0, 534.1, 534.2, 534.3, 534.4, 534.5, 534.6, 534.7, 534.9; |

* The *Any Tumor or Metastatic Tumor*, *Leukemia*, and *Lymphoma* variables were merged in the analytical data set into a single variable, *Metastatic Cancer*.

**S2-5. Staged PCI Identification Algorithm**

Patients who undergo staged PCI do not complete their planned course of treatment at index revascularization. Cardiac Services BC data on staged PCI was incomplete, identifying only seven patients undergoing staged PCI between 2005 and 2011. Therefore, a rule was needed to identify patients with multiple PCI records to differentiate them from patients with repeat revascularization.

Patients who undergo staged PCI do not complete their planned course of treatment at index revascularization. For patients with multiple PCI records having subsequent diagnostic catheterization dates the same or within one Sunday of the one at index, then the date revascularization is achieved is the date of the last PCI if it falls within 60 days of index PCI and 30 days of the non-index PCI. For any unresolved records, we review each record to determine if a last staged PCI date can be identified using the diagnostic catheterization date, the PCI date, and the event type. Any records remaining after the review are considered unresolved and are set aside.

**S2-6. Repeat Revascularization Algorithm**

Repeat revascularization will be identified by the first CABG or PCI occurring after live discharge from index revascularization, when a corresponding DC date differs from the DC date for the index revascularization. In addition, repeat revascularization with PCI will follow index PCI after 60 days or non-index PCI after 30 days. Repeat revascularization is identified using CSBC records.

**S2-7. STROBE Checklist for Cohort Studies**

|  | Item No | Recommendation |  | Reported On Page #^†^ |  |
| --- | --- | --- | --- | --- | --- |
| **Title and abstract** | 1 | (*a*) Indicate the study’s design with a commonly used term in the title or the abstract |  | 1 |  |
|  |  | (*b*) Provide in the abstract an informative and balanced summary of what was done and what was found |  | 2 |  |
| Introduction | | |  |  |  |
| Background/rationale | 2 | Explain the scientific background and rationale for the investigation being reported |  | 4 |  |
| Objectives | 3 | State specific objectives, including any prespecified hypotheses |  | 4 |  |
| Methods | | |  |  |  |
| Study design | 4 | Present key elements of study design early in the paper |  | 5 |  |
| Setting | 5 | Describe the setting, locations, and relevant dates, including periods of recruitment, exposure, follow-up, and data collection |  | 5 |  |
| Participants | 6 | (*a*) Give the eligibility criteria, and the sources and methods of selection of participants. Describe methods of follow-up |  | 6 |  |
|  |  | (*b*) For matched studies, give matching criteria and number of exposed and unexposed |  | N/A |  |
| Variables | 7 | Clearly define all outcomes, exposures, predictors, potential confounders, and effect modifiers. Give diagnostic criteria, if applicable |  | 6, 7, Supplementary Material |  |
| Data sources/ measurement | 8* | For each variable of interest, give sources of data and details of methods of assessment (measurement). Describe comparability of assessment methods if there is more than one group |  | 6, 7 |  |
| Bias | 9 | Describe any efforts to address potential sources of bias |  | 8, 9 |  |
| Study size | 10 | Explain how the study size was arrived at |  | 9, 10 |  |
| Quantitative variables | 11 | Explain how quantitative variables were handled in the analyses. If applicable, describe which groupings were chosen and why |  | 6, 7, 8 |  |
| Statistical methods | 12 | (*a*) Describe all statistical methods, including those used to control for confounding |  | 8, 9 |  |
|  |  | (*b*) Describe any methods used to examine subgroups and interactions |  | N/A |  |
|  |  | (*c*) Explain how missing data were addressed |  | N/A |  |
|  |  | (*d*) If applicable, explain how loss to follow-up was addressed |  | N/A |  |
|  |  | (*e*) Describe any sensitivity analyses |  | N/A |  |
| Results | | |  |  |  |
| Participants | 13* | (a) Report numbers of individuals at each stage of study—eg numbers potentially eligible, examined for eligibility, confirmed eligible, included in the study, completing follow-up, and analysed |  | 10 |  |
|  |  | (b) Give reasons for non-participation at each stage |  | 10, Figure 1 |  |
|  |  | (c) Consider use of a flow diagram |  | 10, Figure 1 |  |
| Descriptive data | 14* | (a) Give characteristics of study participants (eg demographic, clinical, social) and information on exposures and potential confounders |  | 10, 15,  Table 2 |  |
|  |  | (b) Indicate number of participants with missing data for each variable of interest |  | Table 2 |  |
|  |  | (c) Summarise follow-up time (eg, average and total amount) |  | 8 |  |
| Outcome data | 15* | Report numbers of outcome events or summary measures over time |  | 15, 16 |  |
| Main results | 16 | (*a*) Give unadjusted estimates and, if applicable, confounder-adjusted estimates and their precision (eg, 95% confidence interval). Make clear which confounders were adjusted for and why they were included |  | 15, 16 |  |
|  |  | (*b*) Report category boundaries when continuous variables were categorized |  | Table 2 |  |
|  |  | (*c*) If relevant, consider translating estimates of relative risk into absolute risk for a meaningful time period |  | N/A |  |
| Other analyses | 17 | Report other analyses done—eg analyses of subgroups and interactions, and sensitivity analyses |  | Supplemental Material |  |
| Discussion | | |  |  |  |
| Key results | 18 | Summarise key results with reference to study objectives |  | 17 |  |
| Limitations | 19 | Discuss limitations of the study, taking into account sources of potential bias or imprecision. Discuss both direction and magnitude of any potential bias |  | 21 |  |
| Interpretation | 20 | Give a cautious overall interpretation of results considering objectives, limitations, multiplicity of analyses, results from similar studies, and other relevant evidence |  | 22 |  |
| Generalisability | 21 | Discuss the generalisability (external validity) of the study results |  | 22 |  |
| Other information | | |  |  |  |
| Funding | 22 | Give the source of funding and the role of the funders for the present study and, if applicable, for the original study on which the present article is based |  | N/A |  |

^†^Page numbers in this column refer to the original manuscript submission and may vary from the final published paper.

**S2-8. CSBC Data Set Background**

The CSBC database is a prospective registry of cardiovascular procedures. CSBC, a program of the Provincial Health Services Authority, has province-wide responsibility for planning, funding, and evaluating cardiovascular services in British Columbia, a western Canadian province serving approximately five million residents. All coronary revascularization procedures are recorded and stored in the database. Reporting is mandatory, subject to local audit processes, generates operative reports and assists billing. As such, procedural capture is considered near complete. Coronary revascularization occurs across five publicly funded centres. Access to care is universal and based solely on physician assessment of need rather than ability to pay. All patients are entered into the database without requirement for informed consent, therefore avoiding participation bias.

**S2-9. Data Source Citations**

Access to data provided by the Data Steward(s) is subject to approval but can be requested for research projects through the Data Steward(s) or their designated service providers. All inferences, opinions, and conclusions drawn in this publication are those of the author(s), and do not reflect the opinions or policies of the Data Steward(s)

Cardiac Services BC [creator] (2016): Cardiac Services BC Cardiac Registries. Cardiac Services BC [publisher]. Data Extract. CSBC (2018).

Canadian Institute for Health Information [creator] (2018): Discharge Abstract Database (Hospital Separations). V2. Population Data BC [publisher]. Data Extract. MOH (2018).

British Columbia Ministry of Health [creator] (2018): Vital Events Deaths. V2. Population Data BC [publisher]. Data Extract. MOH (2018).

British Columbia Ministry of Health [creator] (2018): Consolidation File (MSP Registration & Premium Billing). V2. Population Data BC [publisher]. Data Extract. MOH (2018).

**S2-10. References**

1. Sobolev B, Levy A, Hayden R, Kuramoto L. Does wait-list size at registration influence time to surgery? Analysis of a population-based cardiac surgery registry. Health services research. 2006 Feb;41(1):23–39.

2. Cottrell KM. Waiting lists: some problems of definition and a relative measure of waiting time. Hosp Heal Serv Rev. 1980;76(8):265–9.

3. Graham MM, Knudtson ML, O’Neill BJ, Ross DB, Group CCSA to CW. Treating the right patient at the right time: Access to cardiac catheterization, percutaneous coronary intervention and cardiac surgery. Canadian Journal of Cardiology. 2006 Jun;22(8):679–83.
